# Prevalence and associated factors of adolescent tobacco use in three Sub-Saharan African countries: A comparative analysis of national cross-sectional surveys

**DOI:** 10.1371/journal.pone.0356891

**Published:** 2026-08-31

**Authors:** Grace Kyule, Samuel Iddi, Noreen Dadirai Mdege, Lyagamula Kisia, Terefe Gelibo Argefa, Olatunbosun Abolarin, Didier M. Mirindi, Retselisitsoe Pokothoane, Boscow Okumu, Nelson Mbaya, Franklin Koech, James Kavai, Akinsewa Akiode, Thompson Ademola, Uche Okezie, Fakorede J.I, Christelle Tchoupé, Damazo T. Kadengye, Shukri F. Mohamed

**Affiliations:** 1 Data Synergy and Evaluation Unit, African Population and Health Research Center, Manga Close, Nairobi, Kenya; 2 Department of Statistics and Actuarial Science, University of Ghana, Accra, Ghana; 3 University of York, Heslington, York, United Kingdom; 4 Centre for Research in Health and Development, York, United Kingdom; 5 Chronic Disease Management Unit, African Population and Health Research Center, Manga Close, Nairobi, Kenya; 6 Development Gateway: An IREX Venture, Washington, District of Columbia, United States of America; 7 Division of Cardiology, University of Ottawa Heart Institute, Ottawa, Canada; 8 Ottawa Model for Smoking Cessation, University of Ottawa Heart Institute, Ottawa, Canada; 9 APIN Public Health Initiatives, Plot 1551, Zone E, Apo Resettlement, FCT, Abuja, Nigeria; 10 Research Initiatives for Social Development, Bukavu, Democratic Republic of the Congo; 11 Research Unit on the Economics of Excisable Products (REEP), School of Economics, University of Cape Town, Cape Town, South Africa; 12 Research and Communications Services Ltd (RCS), Lagos, Nigeria; All India Institute of Medical Sciences, INDIA

## Abstract

Tobacco use often begins during adolescence, increasing the risk of lifelong nicotine dependence and future non-communicable diseases. In sub-Saharan Africa, concerns are rising due to aggressive tobacco industry marketing and expanding nicotine product markets, yet nationally representative data covering both in-school and out-of-school adolescents remain limited. Understanding tobacco use patterns during early and mid-adolescence, a critical period for experimentation and initiation, is essential for informing prevention strategies. We conducted nationally representative cross-sectional household surveys between March and June 2024 using harmonized tools and multistage stratified cluster sampling in Democratic Republic of Congo (DRC), Nigeria and Kenya. Adolescents aged 10–17 years completed standardized face-to-face interviews with response rates of 96% in DRC, 94% in Nigeria and 96% in Kenya. The outcomes were current use of any tobacco, smoked tobacco and smokeless tobacco. Current tobacco use was defined as self-reported use in the past 30 days of any tobacco product including cigarettes, cigars, shisha/waterpipe and smokeless-tobacco (snuff/chewing). Analyses incorporated sampling weights to estimate prevalence, assessed subgroup differences using Rao-Scott chi-square tests, and multivariable logistic regression models were fitted separately for each country and outcome. Among 18,612 adolescents (4,675 in DRC, 7,502 in Nigeria, 6,435 in Kenya), 6·5% (95% CI: 4·9–8·6) reported current tobacco use, with variations across countries (11·9% in DRC, 3·6% in Nigeria, and 2·5% in Kenya). Similar patterns were observed for smoked and smokeless tobacco use. Tobacco use was higher among boys, older adolescents, and those engaged in work. In adjusted analyses, being a boy and older was associated with higher odds of use. Being enrolled in school was linked to lower odds of use in DRC and Kenya, while having both parents alive reduced odds of use in DRC and Nigeria. Higher parental education was associated with lower odds of tobacco use in Kenya but higher odds of use in DRC and Nigeria. Overall, adolescents’ tobacco use remains a critical public health challenge and observed cross-country differences highlight the need for tailored, context-specific interventions and strengthened tobacco control efforts.

## Introduction

Tobacco use during adolescence is a major public health concern, as most adult smokers initiate use before the age of 18 years [1–4], and early initiation increases the likelihood of nicotine dependence, prolonged tobacco use and subsequent tobacco-related diseases later in life [3,5,6]. Globally, an estimated 12% of adolescents aged 13–15 years old use some form of tobacco [7,8]. While tobacco use has declined in many high-income countries, the overall prevalence of tobacco use among adolescents remains high in low- and middle-income countries (LMICs) [7]. LMICs, particularly in sub-Saharan Africa (SSA), are at risk of a rising adolescent tobacco epidemic, driven by a rapidly expanding adolescent population and increasing tobacco industry marketing and investment, exposing adolescents to a variety of products including smoked tobacco (e.g., cigarettes and shisha) and smokeless tobacco (e.g., snuff and chewing tobacco) [9].

Tobacco use is a major risk factor for several non-communicable diseases, including cardiovascular disease, chronic respiratory diseases such as chronic obstructive pulmonary disease, and multiple cancers including lung cancer among others [10–13]. These conditions contribute to more than 8 million tobacco-related deaths annually worldwide, with over 80% of these deaths occurring in LMICs. Without stronger regulatory measures, tobacco attributable deaths in these countries are projected to reach 8.3 million annually by 2030, including 6.8 million in LMICs [14]. Preventing early initiation and addressing tobacco uptake during adolescence are therefore critical steps toward reducing the long-term burden of tobacco-related morbidity and mortality in Africa [15,16]. Despite this urgency, data on adolescent tobacco use in SSA is limited, hindering the development of targeted, evidence-based regulatory and control strategies to prevent initiation and prevent use among young populations.

Although many countries have conducted the Global Youth Tobacco Surveys (GYTS) and the Global School-based Health Survey (GSHS), these surveys are implemented at irregular intervals and primarily include adolescents attending school. Consequently, nationally representative evidence describing tobacco use among both in-school and out-of-school adolescents remains limited, reducing the ability to generate population-wide estimates and identify vulnerable subgroups. Adolescents who are not enrolled in school may also experience different social and environmental exposures associated with tobacco use, underscoring the importance of including them in nationally representative surveys [17–20]. To address this evidence gap, we conducted nationally representative household-based surveys to obtain data on tobacco use among adolescents as part of the Data on Youth and Tobacco (DaYTA) program in three countries; the Democratic Republic of the Congo (DRC), Nigeria, and Kenya. Each country has adopted tobacco control measures to varying extents, offering a unique opportunity to compare patterns of adolescent tobacco use across diverse regions with variations in tobacco control policy implementation. In this paper, we present pooled and country-specific prevalence estimates as well as correlates of tobacco use among adolescents in the three countries.

## Methods

### Study design and setting

We conducted nationally representative cross-sectional household surveys of adolescents in three countries: the DRC, Nigeria, and Kenya. Data collection took place during the following periods: the DRC (27 March 2024 to 17 June 2024), Nigeria (03 May 2024 to 30 June 2024) and Kenya (05 April 2024 to 21 May 2024). These dates represent the full recruitment window for each study site. To ensure cross-country comparability, all three country surveys employed a harmonized, structured questionnaire developed as part of the DaYTA program. The questionnaire was informed by established tobacco surveillance instruments, including modules adapted from the GYTS, GSHS, Center for Disease Control (CDC) National Youth Tobacco Survey (NYTS), the ASH Smokefree Great Britain Youth survey (ASH-Y) and the WHO Tobacco Questions for Surveys of Youth (TQS-Youth), and was reviewed by tobacco control and adolescent health experts across the three study countries. The tool was piloted during preparatory field testing to ensure clarity, cultural appropriateness, and consistency across settings.

Standardized sampling methodologies were implemented across countries. In each country, a multistage stratified cluster sampling design was used. In the first stage, administrative regions or provinces were selected to ensure geographic representation. Within these regions, enumeration areas (EAs) were randomly selected using probability proportional to size based on the most recent national census sampling frames. Households within the selected EAs were then systematically sampled. In households with at least one eligible adolescent aged 10–17 years, one adolescent was randomly selected to participate in the survey. The overall response rates among eligible adolescents were 96% in the DRC, 94% in Nigeria, and 96% in Kenya.

In the DRC, data was collected from 144 local clusters classified as villages in rural areas or avenues in urban areas, which served as the enumeration areas (EAs) across 16 randomly selected provinces. In Nigeria, data were collected from 265 enumeration areas (EAs) in 12 states and the Federal Capital Territory, covering all six geopolitical zones. In Kenya, 224 EAs were sampled from 16 counties across all former provincial regions. All variables and methods were defined uniformly across the three countries to ensure comparability in pooled and country-specific analyses. Written informed consent was obtained from parents or guardians of all participating minors, and written assent was obtained from adolescents prior to data collection. A detailed description of the DaYTA survey methodology is available in a published protocol [21].

### Measures

For this paper, the primary outcome was the prevalence of current tobacco use, defined as the use of any tobacco product within the past 30 days. This outcome was estimated across three tobacco products categories namely; (i) any tobacco use, which comprises the use of any form of tobacco, either smoked, heated or smokeless, (ii) smoked tobacco, which referred to combustible products such as manufactured or hand-rolled cigarettes, shisha, cigars, pipes, or local smoked products and (iii) smokeless tobacco, which entails non-combustible forms such as chewing tobacco, snuff, or snus. These definitions were consistent with standard surveillance definitions of youth tobacco use.

Several explanatory variables were examined as potential correlates of tobacco use based on prior evidence from existing literature and a socio-ecological framework of adolescent tobacco use [22,23]. Individual-level factors included age categorized as 10–12, 13–15 and 16–17 years, sex (boys or girls), current schooling status (in-school vs out-of-school), and engagement in any form of work (self-employed or employed). Out-of-school adolescents were defined as those who were not enrolled in formal education, including those who had never attended school or who had previously enrolled but were not currently attending school at the time of the survey, either because they had dropped out or had completed secondary schooling but were not enrolled in college or another educational institution. Adolescents who had completed secondary school represented only 0.3% of the pooled study population. Given the very small size of this subgroup, separate analyses would have produced unstable estimates and limited interpretability. Therefore, these adolescents were classified within the out-of-school category to reflect their non-enrolment in formal education at the time of data collection.

Household-level factors comprised the sex of the household head (male or female), the household head’s education level (secondary school or higher vs. less than secondary) and wealth quintile which was constructed using principal component analysis based on household assets, housing characteristics, and access to utilities, and households were categorized into quintiles from lowest to highest socioeconomic status. We also included whether both biological parents were reported to be alive as a proxy for orphan status or potential family disruption. Environmental factors included place of residence (urban vs rural) and exposure to smoking in public spaces which was defined as having seen anyone smoking on school premises, universities or health facilities buildings or in public transport in the past 30 days.

Adolescents’ functional disability was assessed using the Washington Group Short Set (WG-SS) of Child Functioning questions and categorized as none, mild, moderate, or severe. This variable was presented in the descriptive characteristics of the study population but was not included in the regression analyses due to small numbers in some categories, which could affect the stability of the model estimates.

### Statistical analysis

Data was analysed using Stata version 18, accounting for the complex survey design in all calculations by applying sampling weights and using the survey (*svy)* commands to adjust for clustering and stratification. All estimated results are representative of the national adolescent population in each country. We first computed descriptive statistics and prevalence estimates. Weighted prevalences of current use of any tobacco, smoked tobacco, and smokeless tobacco were calculated for each country separately, as well as for the pooled sample. For the pooled estimates, country-specific weights were applied without normalization, allowing each country to contribute proportionally based on its population size and thus reflecting the overall population burden of tobacco use across the three countries. All prevalence estimates are presented with corresponding 95% confidence intervals (CIs). We further examined subgroup differences in current tobacco use within each country and across the countries. Prevalence of current use of any tobacco, and by product type was stratified by sex, age group, school status, place of residence, employment status, and orphan status.

To identify factors associated with current tobacco use, we conducted multivariable logistic regression analyses. Separate models were estimated for each country and each of the three outcome variables; current use of any tobacco, smoked tobacco and smokeless tobacco. Each model included a set of explanatory variables representing individual-, household- and environmental- level explanatory variables. Adjusted odds ratios (aORs) with 95% CIs were estimated for each explanatory variable while controlling for all other variables included in the model. Statistical significance was evaluated at the 5% level. We checked model fit and multicollinearity assumptions and no concerning issues were found as all variance inflation factors were < 2, and goodness-of-fit tests were satisfactory. Missing data were minimal (<5% across variables). Responses recorded as “refused” were treated as missing and excluded from regression analyses using a complete-case approach.

#### Ethical considerations.

Ethics approvals were obtained from review boards in each country: the National Health Ethics Committee in the DRC (513/CNES/BN/PMMF/2024_17/02/2024), the National Health Research Ethics Committee (NHREC) in Nigeria (NHREC/01/01/2007–23/01/2024), and AMREF Ethics and Scientific Review Committee (ESRC) in Kenya (ESRC P1570/2023). Written parental consent and adolescent assent were obtained prior to participation in each country. All data was anonymized, and interviews were conducted privately to ensure confidentiality.

#### Inclusivity in global research.

Additional information regarding the ethical, cultural and scientific considerations specific to inclusivity in global research is included in the Supporting Information (S1 Checklist).

## Results

### Descriptive characteristics

A total of 18,612 adolescents (4,675 in the DRC, 7,502 in Nigeria and 6,435 in Kenya) participated in the study and were included in the analysis across the three countries. Table 1 summarizes the background characteristics of the study population. In the pooled sample, sex distribution was nearly equal, with 51.2% boys and 48.8% girls. Most adolescents were aged 10–12 years (42.9%), and this distribution was consistent across the three countries. Most adolescents were enrolled in school at the time of the survey, with 84.5% reporting that they were currently enrolled in school. Kenya had the highest proportion of in-school adolescents (91.0%). Overall, 65.2% of adolescents resided in rural areas, with rural residence most common in the DRC (79.9%) and Kenya (76.6%). In Nigeria, the urban–rural distribution was more balanced, with 51.2% of adolescents living in rural areas. Over ten percent (11.9%) of the pooled sample reported being employed or self-employed. Kenya had the lowest proportion of adolescents engaged in work (6.7%), while the DRC had the highest (14.4%).

**Table 1:** Background characteristics of adolescents in the pooled sample and by country.

Background characteristics	Pooled sample % (95% CI)	Nigeria % (95% CI)	Kenya % (95% CI)	DRC % (95% CI)
**Overall**	100	40.31 (36.98, 43.72)	34.57 (31.70-37.56)	25.11 (20.09, 30.92)
**Sex**				
Boys	51.19 (50.27, 53.60)	52.21 (50.24, 54.18)	48.69 (46.92-50.46)	52.72 (49.01-56.40)
Girls	48.81 (46.40, 49.73)	47.79 (45.82, 49.76)	51.31 (49.53,53.07)	47.28 (43.60, 50.99)
**Age group**				
10-12	42.87 (40.77, 44.99)	42.54 (39.36, 45.77)	39.92 (37.19, 42.72)	44.34 (40.81, 47.93)
13-15	36.08 (34.30, 37.99)	35.02 (32.03, 38.14)	37.63 (34.72, 40.63)	36.94 (34.61, 39.33)
16-17	21.05 (19.58, 22.61)	22.44 (19.89, 25.21)	22.45 (20.63, 24.38)	18.72 (16.96, 20.61)
**School status †**				
Out of school	15.54 (13.70, 17.58)	19.45 (17.14, 21.99)	8.99 (6.65, 12.05)	12.24 (9.38, 15.81)
In school	84.46 (82.41, 86.30)	80.26 (77.71, 82.59)	90.96 (87.90, 93.30)	87.76 (84.19, 90.62)
**Residence †**				
Rural	65.24 (60.35, 69.83)	51.24 (47.97, 54.50)	76.63 (74.59, 78.54)	79.87 (70.12, 87.04)
Urban	34.76 (30.17, 39.65)	48.76 (45.50, 52.03)	23.37 (21.46, 25.41)	20.12 (12.96, 29.88)
**Employment status †**				
Not employed	88.22 (86.53, 89.73)	88.68 (87.13, 90.07)	93.35 (92.32, 94.25)	85.64 (81.89, 88.73)
Employed	3.73 (2.39, 5.76)	2.90 (2.18, 3.84)	4.92 (4.14, 5.84)	4.46 (1.72, 11.05)
Self-employed	8.05 (7.17, 9.02)	8.42 (7.19, 9.85)	1.73 (1.33, 2.26)	9.90 (7.78, 12.53)
**Orphan status (having both parents alive)**				
No	14.22 (12.98, 15.56)	12.04 (10.84, 13.34)	17.40 (16.13, 18.74)	13.35 (9.57, 18.33)
Yes	85.78 (84.44, 87.02)	87.96 (86.66, 89.16)	82.60 (81.26, 83.87)	86.65 (81.67, 90.43)
**Functional disability**				
None	86.08 (84.51, 87.52)	90.80 (89.36, 92.06)	82.55 (80.94, 84.05)	81.05 (77.72, 83.99)
Mild	11.00 (9.90, 12.21)	7.84 (6.72, 9.12)	15.06 (13.72, 16.52)	13.79 (11.31, 15.70)
Moderate	2.54 (1.65, 3.90)	1.23 (0.88, 1.73)	1.96 (1.57, 2.45)	4.48 (2.25, 8.75)
Severe	0.37 (0.15, 0.92)	0.13 (0.05, 0.31)	0.43 (0.25, 0.75)	0.68 (0.16, 2.77)
**Wealth quintile**				
Poorest	23.50 (21.51, 25.62)	29.22 (25.80, 32.52)	24.84 (21.61, 28.58)	19.44 (12.11, 29.71)
Poor	20.99 (19.43, 22.44)	22.23 (20.32, 24.56)	20.77 (19.07, 22.44)	20.16 (15.56, 25.72)
Middle	19.60 (17.56, 21.80)	16.67 (15.40, 19.03)	19.93 (18.00, 21.56)	21.67 (17.91, 25.98)
Rich	18.22 (17.09, 19.40)	15.03 (13.97, 17.44)	18.04 (16.28, 20.43)	19.67 (16.56, 23.20)
Richest	17.79 (15.09, 20.85)	15.85 (13.92, 17.90)	16.41 (14.84, 18.05)	19.04 (9.34, 34.94)

Note: Values are survey-weighted percentages with 95% confidence intervals and account for the complex survey design.

† Indicates statistically significant differences in subgroup distribution across countries based on the Rao–Scott corrected Pearson χ² test (p < 0.05).

CI = confidence interval; DRC = Democratic Republic of the Congo.

### Current use prevalence of any tobacco

The pooled prevalence of current any tobacco use among adolescents across the three countries was 6.5%, with variation by country (Table 2). The Democratic Republic of Congo reported a significantly higher prevalence at 11.9% compared to Nigeria (3.6%) and Kenya (2.5%), whose prevalence rates were statistically not different. Across countries, boys had a higher prevalence of any tobacco use than girls, and any tobacco use increased with age, except in Kenya where the highest prevalence was observed among adolescents aged 13–15 years. Among adolescents attending school, prevalence was lower, especially in Kenya, where only 1.0% reported current use. In contrast, tobacco use among out-of-school adolescents in Kenya was higher at 17.5%. In the pooled sample, tobacco use was more than twice as high among employed or self-employed than non-employed adolescents, a pattern also observed in Nigeria. Prevalence was also higher among adolescents who had lost one or both parents, with significant differences in Nigeria and the DRC.

**Table 2:** Prevalence of any tobacco use among adolescents.

Characteristic	Pooled % (95% CI)	Nigeria % (95% CI)	Kenya % (95% CI)	DRC % (95% CI)
Current use	6.54 (4.93, 8.63)	3.64 (2.94, 4.49)	2.47 (1.51, 4.02)	11.85 (6.92, 19.55)
**Sex**				
Boys	9.17 (7.01, 11.93) †	5.38 (4.28, 6.73) †	3.18 (2.01, 5.02) †	16.13 (9.92, 25.14) †
Girls	3.70 (2.62, 5.20)	1.74 (1.16, 2.58)	1.80 (0.97, 3.31)	7.07 (3.74, 12.97)
**Age group**				
10-12	4.62 (3.09, 6.86) †	2.08 (1.32, 3.25) †	1.33 (0.68, 2.60)	8.91 (4.94, 15.57)
13-15	6.64 (4.88, 8.96)	3.43 (2.45, 4.76)	3.60 (1.55, 8.16)	11.78 (6.70, 19.86)
16-17	10.30 (7.71, 13.63)	6.92 (4.85, 9.78)	2.61 (1.75, 3.86)	18.94 (10.18, 32.51)
**Residence**				
Rural	6.69 (4.88, 9.12)	3.71 (2.78, 4.93)	2.75 (1.55, 4.82)	10.58 (5.86, 18.36)
Urban	6.26 (4.56, 8.54)	3.56 (2.60, 4.86)	1.57 (0.89, 2.75)	16.90 (9.84, 27.47)
**Schooling status**				
In school	6.10 (4.58, 8.08) †	3.44 (2.65, 4.45)	0.98 (0.69, 1.39) †	11.22 (6.58, 18.47)
Out of school	8.94 (6.51, 12.19)	4.45 (3.22, 6.13)	17.49 (9.59, 29.76)	16.38 (9.39, 27.00)
**Employment status**				
Not employed	5.71 (4.20, 7.71)	2.97 (2.29, 3.84) †	2.32 (1.33, 4.03)	11.10 (6.24, 18.99)
Employed	13.73 (9.94, 18.65)	15.60 (9.11, 25.43)	4.13 (2.14, 7.82)	15.98 (9.24, 26.21)
Self employed	12.88 (7.71, 20.76)	6.36 (4.16, 9.60)	5.74 (2.27, 13.74)	21.20 (7.00, 49.04)
**Orphan status (both parents alive)**				
No	10.83 (6.93, 16.55) †	6.13 (3.47, 10.59) †	2.46 (1.31, 4.56)	18.92 (9.88, 22.16) †
Yes	5.82 (4.41, 7.64)	3.05 (2.43, 3.83)	2.44 (1.40, 4.21)	10.70 (6.22, 17.80)

Note: All estimates are survey-weighted and account for the complex survey design.

† Indicates a statistically significant difference between subgroups within each country and in the pooled sample based on a design-adjusted F-test from the Rao–Scott corrected Pearson chi-square test.

Smoked tobacco use among adolescents was highest in the DRC (7.98%) and lowest in Kenya (1.03%), with a pooled prevalence of 4.52%. Boys had a higher pooled prevalence of smoked tobacco use than girls (6.98% vs. 1.85%), a pattern consistent across all three countries. Older adolescents (13–15 years and 16–17 years) reported higher use than younger ones (10–12 years), with significant differences seen both in the pooled data and across countries. Out-of-school adolescents had significantly higher use than their in-school counterparts in the pooled estimates, Kenya, and the DRC. Although urban adolescents reported more use than rural ones, a significant urban-rural gap was found only in the DRC (Table 3).

**Table 3:** Prevalence of smoked tobacco use among adolescents.

Characteristic	Pooled % (95% CI)	Nigeria % (95% CI)	Kenya % (95% CI)	DRC % (95% CI)
Current use	4.52 (3.24, 6.26)	2.84 (2.28, 3.53)	1.03 (0.69, 1.53)	7.98 (4.23, 14.55)
**Sex**				
Boys	6.98 (5.09, 9.51) †	4.18 (3.27, 5.32) †	1.57 (1.06, 2.32) †	12.44 (7.12, 20.84) †
Girls	1.85 (1.22, 2.81)	1.38 (0.85, 2.23)	0.52 (0.22, 1.21)	3.00 (1.30, 6.79)
**Age group**				
10-12	2.65 (1.63, 4.27) †	1.33 (0.81, 2.18) †	0.33 (0.12, 0.88)	5.06 (2.47, 10.07) †
13-15	4.75 (3.35, 6.68)	2.81 (1.92, 4.11)	1.31 (0.65, 2.60)	8.42 (4.46, 15.35)
16-17	7.93 (5.70, 10.94)	5.74 (4.12, 7.95)	1.81 (1.15, 2.82)	14.02 (6.81, 26.69)
**Residence**				
Rural	4.16 (2.81, 6.11)	2.28 (1.64, 3.13)	1.01 (0.62, 1.63)	6.84 (3.47, 13.05) †
Urban	5.18 (3.84, 6.96)	3.43 (2.55, 4.61)	1.10 (0.58, 2.08)	12.50 (6.95, 21.46)
**Schooling status**				
In school	4.10 (2.97, 5.65) †	2.72 (2.09, 3.53)	0.60 (0.39, 0.93) †	7.06 (3.79, 12.77) †
Out of school	6.76 (4.55, 9.94)	3.31 (2.24, 4.88)	5.32 (2.64, 10.40)	14.54 (7.80, 25.49)
**Employment status**				
Not employed	3.74 (2.56, 5.44) †	2.24 (1.72, 2.90) †	0.91 (0.57, 1.44) †	7.10 (3.48, 13.92) †
Employed	11.32 (8.30, 15.26)	13.57 (7.11, 24.35)	2.58 (1.07, 6.07)	12.88 (10.52, 15.67)
Self employed	9.96 (5.70, 16.83)	5.25 (3.24, 8.41)	3.13 (1.05, 8.96)	16.07 (4.59, 43.21)
**Orphan status (both parents alive)**				
No	8.47 (4.83, 14.43) †	4.83 (2.41, 9.44) †	0.63 (0.24, 1.67)	15.20 (6.78, 30.68) †
Yes	3.88 (2.82, 5.32)	2.31 (1.80, 2.93)	1.00 (0.62, 1.61)	6.98 (3.75, 12.63)

Note: All estimates are survey-weighted and account for the complex survey design.

† Indicates a statistically significant difference between subgroups within each country and in the pooled sample based on a design-adjusted F-test from the Rao–Scott corrected Pearson chi-square test.

Smokeless tobacco use among adolescents was highest in the DRC and lowest in Nigeria, with a pooled prevalence of 3.0% (Table 4). Boys reported higher use than girls in the pooled sample, with significant differences observed in Nigeria. Use increased slightly with age in the DRC. Out-of-school adolescents had higher prevalence than those in school, with the most striking difference seen in Kenya, where out-of-school use was over 15% compared to less than 1% among those in school. In the pooled data, adolescents who had lost one or both parents had higher smokeless tobacco use than those with both parents alive (5.10% vs. 2.69%), with significant differences in Nigeria. Rural use was higher than urban use in Kenya.

**Table 4:** Prevalence of Smokeless Tobacco Use Among Adolescents.

Characteristic	Pooled % (95% CI)	Nigeria % (95% CI)	Kenya % (95% CI)	DRC % (95% CI)
Current use	2.95 (2.09, 4.16)	1.10 (0.73, 1.64)	1.72 (0.94, 3.13)	5.86 (3.42, 9.87)
**Sex**				
Boys	3.65 (2.52, 5.25) †	1.72 (1.05, 2.78) †	2.05 (1.18, 3.52)	6.71 (3.81, 11.56)
Girls	2.21 (1.50, 3.23)	0.42 (0.26, 0.68)	1.41 (0.65, 3.00)	4.91 (2.78, 8.54)
**Age group**				
10-12	2.75 (1.77, 4.25) †	1.12 (0.52, 2.41)	1.19 (0.57, 2.47)	5.31 (2.86, 9.67) †
13-15	2.96 (1.91, 4.55)	0.90 (0.55, 1.46)	2.75 (1.08, 6.86)	5.63 (2.89, 10.69)
16-17	3.37 (2.43, 4.65)	1.35 (0.71, 2.58)	0.92 (0.46, 1.82)	7.61 (4.66, 12.21)
**Residence**				
Rural	3.28 (2.41, 4.44)	1.76 (1.26, 2.46)	2.07 (1.08, 3.94) †	4.99 (2.87, 8.54)
Urban	2.34 (1.18, 4.57)	0.40 (0.00, 2.10)	0.56 (0.25, 1.26)	9.33 (4.17, 19.57)
**Schooling status**				
In school	2.86 (2.01, 4.05)	1.04 (0.64, 1.69)	0.37 (0.22, 0.64) †	5.97 (3.53, 9.92)
Out of school	3.48 (1.96, 6.13)	1.31 (0.63, 2.66)	15.23 (8.16, 26.67)	5.10 (1.67, 14.50)
**Employment status**				
Not employed	2.86 (1.76, 4.62)	1.02 (0.63, 1.63)	1.69 (0.87, 3.24)	6.05 (3.38, 10.61)
Employed	3.08 (1.12, 8.23)	3.10 (0.85, 10.69)	1.55 (0.58, 4.08)	3.70 (0.49, 23.05)
Self employed	4.36 (1.92, 9.58)	1.29 (0.66, 2.52)	3.85 (1.23, 11.43)	8.07 (3.32, 18.34)
**Orphan status (both parents alive)**				
No	5.10 (2.51, 10.09) †	3.07 (1.18, 7.74) †	1.74 (0.76, 3.94)	8.48 (3.12, 21.06)
Yes	2.69 (1.95, 3.71)	0.92 (0.63, 1.34)	1.79 (0.94, 3.36)	5.40 (3.19, 8.98)

Note: All estimates are survey-weighted and account for the complex survey design.

† Indicates a statistically significant difference between subgroups within each country and in the pooled sample based on a design-adjusted F-test from the Rao–Scott corrected Pearson chi-square test.

### Adjusted multivariable factors associated with tobacco use

Table 5 presents adjusted odds ratios (aORs) from country-specific multivariable logistic regression models for current use of any tobacco product, smoked tobacco, and smokeless tobacco. Across all three countries, boys had significantly higher odds of any tobacco use than girls, with the odds being about two times high in Nigeria (aOR = 2.15, 95% CI 1.56–2.88), Kenya (aOR = 2.05, 95% CI 1.31–3.24), and DRC (aOR = 2.47, 95% CI 1.63–3.75) Table 5. This association was consistent across both smoked and smokeless tobacco.

**Table 5:** Adjusted factors associated with current use of any tobacco, smoked tobacco and smokeless tobacco among adolescents in Nigeria, Kenya, and the DRC.

	Any tobacco			Smoked tobacco			Smokeless tobacco		
	Nigeria	Kenya	DRC	Nigeria	Kenya	DRC	Nigeria	Kenya	DRC
	aOR (95% CI)	aOR (95% CI)	aOR (95% CI)	aOR (95% CI)	aOR (95% CI)	aOR (95% CI)	aOR (95% CI)	aOR (95% CI)	aOR (95% CI)
**Sex (ref: girl)**									
Boy	2.15 (1.56, 2.88)	2.05 (1.31, 3.24)	2.47 (1.63, 3.75)	1.95 (1.32, 2.88)	2.80 (1.39, 5.65)	4.66 (2.83, 7.67)	1.99 (1.34, 2.94)	1.97 (1.12, 3.48)	1.45 (1.01, 2.11)
**Age group (ref: 10–12 years)**									
13-15	2.48 (1.76, 3.48)	2.21 (1.29, 3.80)	1.28 (0.83, 2.00)	3.55 (2.27, 5.55)	2.08 (0.90, 4.82)	1.67 (1.05, 2.64)	3.58 (2.29, 5.59)	2.14 (1.14, 4.02)	0.94 (0.59, 1.48)
16-17	5.11 (3.52, 7.43)	2.12 (1.10, 4.09)	1.94 (1.14, 3.32)	7.72 (4.80, 12.39)	3.90 (1.58, 9.65)	2.43 (1.30, 4.52)	7.95 (4.96, 12.76)	0.57 (0.21, 1.55)	1.33 (0.97, 1.84)
**Schooling status (ref: out-of-school)**									
In school	1.29 (0.87, 1.92)	0.06 (0.04, 0.09)	0.57 (0.32, 1.01)	1.10 (0.70, 1.75)	0.23 (0.11, 0.48)	0.39 (0.20, 0.77)	1.16 (0.73, 1.84)	0.02 (0.01, 0.04)	1.03 (0.57, 1.87)
**Work engagement (ref: none)**									
Employed	2.79 (1.55, 5.00)	1.43 (0.66, 3.11)	0.74 (0.34, 1.58)	2.71 (1.38, 5.32)	2.25 (0.88, 5.75)	0.72 (0.34, 1.51)	2.80 (1.43, 5.49)	1.10 (0.36, 3.39)	0.66 (0.08, 5.39)
Self employed	2.07 (1.37, 3.12)	1.57 (0.59, 4.12)	1.63 (0.71, 3.73)	2.29 (1.42, 3.70)	3.34 (1.05, 10.61)	1.64 (0.60, 4.51)	2.47 (1.54, 3.95)	1.31 (0.40, 4.27)	1.72 (0.61, 4.84)
**Both parents alive (ref: no)**									
Yes	0.45 (0.32, 0.66)	1.04 (0.55, 1.99)	0.48 (0.25, 0.94)	0.50 (0.33, 0.78)	1.14 (0.44, 2.95)	0.38 (0.17, 0.83)	0.52 (0.33, 0.78)	1.53 (0.62, 3.75)	0.88 (0.43, 1.79)
**Household head sex (ref: female)**									
Male	1.13 (0.83, 1.54)	1.70 (1.10, 2.64)	1.35 (1.01, 1.80)	1.06 (0.73, 1.53)	1.92 (1.02, 3.61)	1.50 (1.25, 1.80)	1.05 (0.73, 1.52)	1.37 (0.14, 1.99)	1.21 (0.76, 1.92)
**Household head education (ref: primary and below)**									
Secondary +	1.69 (1.21, 2.36)	0.58 (0.34, 0.99)	1.74 (1.21, 2.50)	1.77 (1.19, 2.64)	0.65 (0.32, 1.76)	1.39 (1.03, 1.86)	1.71 (1.15, 2.54)	0.37 (0.06, 0.41)	1.62 (0.84, 3.13)
**Residence (ref: urban)**									
Rural	1.32 (0.98, 1.78)	1.71 (1.10, 2.67)	0.74 (0.48, 1.16)	0.74 (0.53, 1.04)	0.77 (0.41, 1.47)	0.62 (0.41, 0.93)	0.72 (0.52,1.02)	2.90 (1.47, 5.71)	0.57 (0.24, 1.38)
**Wealth quintile (ref: poorest)**									
Poor	0.96 (0.63, 1.46)	0.21 (0.10, 0.45)	1.35 (0.77, 2.39)	1.01 (0.60, 1.69)	0.74 (0.31, 1.77)	1.30 (0.60, 2.81)	1.03 (0.61, 1.72)	0.06 (0.01, 0.26)	0.99 (0.61, 1.60)
Middle	0.84 (0.54, 1.32)	0.23 (0.11, 0.49)	0.99 (0.53, 1.87)	0.90 (0.53, 1.54)	0.44 (0.15, 1.26)	0.96 (0.43, 2.13)	0.92 (0.54, 1.58)	0.10 (0.03, 0.34)	0.81 (0.47, 1.39)
Rich	0.53 (0.32, 0.88)	0.47 (0.26, 0.87)	0.88 (0.49, 1.59)	0.64 (0.35, 1.14)	0.90 (0.37, 2.18)	0.98 (0.43, 2.25)	0.68 (0.38, 1.22)	0.19 (0.07, 0.50)	0.52 (0.31, 0.88)
Richest	0.65 (0.39, 1.07)	0.30 (0.14, 0.62)	1.02 (0.58, 1.79)	0.91 (0.51, 1.60)	0.66 (0.24, 1.80)	1.07 (0.49, 2.33)	0.94 (0.53, 1.66)	0.04 (0.01, 0.33)	0.77 (0.39, 1.52)
**Exposure in public places (ref: no)**									
Yes	1.43 (1.02, 1.99)	1.14 (0.72, 1.82)	3.98 (1.53, 10.32)	1.44 (0.97, 2.15)	1.63 (0.83, 3.20)	4.15 (1.30, 13.23)	–	–	–

Note: Adjusted odds ratios (aOR) are derived from survey-weighted multivariable logistic regression models accounting for clustering and stratification. Associations are considered statistically significant when the 95% confidence interval does not include 1.0.

Older adolescents had higher odds of tobacco use across all countries. In Nigeria, the odds of any tobacco use increased with age (aOR = 2.48, 95% CI 1.76–3.48) for ages 13−15 and (aOR = 5.11, 95% CI 3.52–7.43) for 16−17 years compared with 10−12 years with a similar pattern observed for both smoked and smokeless tobacco (Table 5). In Kenya, older adolescents had higher odds of any tobacco use (aOR = 2.21, 95% CI 1.29–3.80 and aOR = 2.12, 95% CI 1.10–4.09 for ages 13–15 and 16–17, respectively) and of smoked tobacco use (aOR = 3.90, 95% CI 1.58, 9.65 for ages 16–17). In the DRC, older age (16−17 years) was similarly associated with higher odds of any tobacco (aOR = 1.94, 95% CI 1.14, 3.32) and smoked tobacco (aOR = 2.43, 95% CI 1.30, 4.52). School attendance was associated with lower odds of smoked tobacco use in Kenya and DRC. Adolescents who were in school had lower odds of any tobacco use (Kenya aOR = 0.06, 95% CI 0.04–0.09), smoked tobacco (Kenya aOR = 0.23, 95% CI 0.11–0.48; DRC aOR = 0.39, 95% CI 0.20–0.77), and smokeless tobacco (Kenya aOR = 0.02, 95% CI 0.01–0.04). No significant associations were found in Nigeria and for smokeless tobacco in DRC.

Being employed or self-employed increased the odds of any tobacco, smoked and smokeless tobacco in Nigeria and self-employment increased the odds of use for smoked tobacco in Kenya. Having both parents alive was associated with lower odds of any and smoked tobacco use in Nigeria and the DRC, and with lower odds of smokeless tobacco use in Nigeria only. Adolescents from households headed by males had higher odds of use for any tobacco and smoked tobacco in Kenya and the DRC. Higher household head education (secondary or more) was linked to lower odds of any tobacco (aOR = 0.58, 95% CI 0.34–0.99) and smokeless tobacco (aOR = 0.37, 95% CI 0.06–0.41) use in Kenya. Conversely, it was associated with significantly higher odds of any form of tobacco use and smoked tobacco in Nigeria and DRC.

Rural residence was linked to increased odds of any tobacco use (aOR = 1.71, 95% CI 1.10–2.67) and smokeless tobacco use (aOR = 2.90, 95% CI 1.47–5.71) in Kenya, and decreased odds in the DRC for smoked tobacco (aOR = 0.62, 95% CI 0.41–0.93). In Kenya, higher wealth was associated with lower odds of tobacco use. Adolescents from richer households had reduced odds of any tobacco and smokeless tobacco use compared to those from poorest households. No consistent pattern by wealth was observed in Nigeria or DRC. Finally, exposure to tobacco use in public places was associated with higher odds of adolescent tobacco use in both the DRC and Nigeria, including increased odds of any tobacco use in the DRC (aOR = 3.98, 95% CI 1.53–10.32) and smoked tobacco use (aOR = 4.15, 95% CI 1.30–13.23).

## Discussion

Tobacco use among adolescents in the DRC, Nigeria and Kenya poses significant public health concerns with long-term implications for non-communicable diseases, increased health system burden, and adverse social outcomes. The pooled prevalence of any tobacco use was 6.5%, with the DRC reporting the highest levels across all forms of tobacco use. Boys and older adolescents had higher odds of tobacco use across countries. Being enrolled in school was associated with lower odds of smoked tobacco use in Kenya and the DRC, while having both parents alive reduced the odds of any tobacco use in Nigeria and the DRC. Higher household head education was linked to lower odds of tobacco use in Kenya but to higher odds in Nigeria and the DRC. Exposure to tobacco in public places increased the odds of use in the DRC and Nigeria. These findings underscore the importance of context-specific interventions that combine school-based retention strategies, family support initiatives, and stricter enforcement of tobacco control policies.

The pooled prevalence of any tobacco use across the three countries (6.5%) fell within the range of 2–8% reported in a SSA systematic review [24]. However, it was lower than the continent estimate of 14.3% from a recent Africa-wide review [25], and the 19.1% reported in a pooled analysis of 22 African countries using GYTS data (2013–2018) [26]. The lower prevalence likely reflects the study’s focus on three countries, inclusion of both in- and out-of-school adolescents, and recent survey periods coinciding with strengthened tobacco control enforcement and increased awareness. Overall, our findings are broadly consistent with WHO-reported declines in tobacco use [7].

Prevalence differences across countries likely reflect variations in the scope, timing of tobacco control legislation, and the broader social and market contexts in which these measures are implemented. Specifically, challenges with enforcement and compliance being evident across all three countries. In the DRC, despite the adoption of the Framework Public Health Law in 2018 and ministerial orders such as the 2022 decree on advertising and smoke-free environments, the continued availability of single sticks, weak restrictions on sales to minors, and the circulation of illicit products that lack tax stamps or health warnings has increased accessibility for adolescents [27–30]. Sociocultural acceptance of tobacco use may also contribute to normalizing experimentation [29], while ongoing political and economic instability in the DRC may have limited consistent implementation of health promotion programs and age-restriction laws. In Kenya and Nigeria, even if tobacco control measures have not been implemented and enforced perfectly, policies and action measures were adopted early. In Kenya, the Tobacco Control Act (2007) and subsequent regulations introduced bans on public smoking and tobacco advertising, restrictions on sales near schools, pictorial health warnings, and higher excise taxes [31], while Nigeria National Tobacco Control Act (2015) included age-of-sale prohibitions, graphic health warnings, and excise tax reforms [32]. These earlier and broader measures may have reduced affordability and visibility of tobacco products among adolescents, which may help explain the comparatively lower prevalence of adolescent tobacco use in Kenya and Nigeria.

As expected, the prevalence of any tobacco use was higher than that of smoked or smokeless tobacco use considered separately; however, product-specific analyses revealed important differences in patterns of use. Smoked tobacco use was particularly high in the DRC, while smokeless tobacco use, though lower overall, also showed meaningful subgroup differences, especially by school status and sex. These findings suggest that adolescents are not exposed to a single, uniform tobacco market. Rather, product availability, cost, social acceptability, and context-specific norms may shape whether young people use smoked or smokeless products. This supports the need for surveillance and prevention strategies that distinguish between tobacco product categories rather than focusing only on aggregate “any tobacco use.”

Adolescents sex and age were associated with tobacco use across the three countries. Boys were significantly more likely than girls to use tobacco both smoked and smokeless products, reflecting widespread gendered norms around tobacco use in many African societies and globally [24,25,33]. This disparity is further reinforced by the tobacco industry’s historical targeting of boys and young men in Africa [34]. However, there are indications that the gender gap may be narrowing, as marketing increasingly targets young women [35]. A study on cigarette packaging across 14 low- and middle-income countries documented the deliberate use of feminine imagery such as floral designs, pink or glittery colors, slim pack shapes, and flavor cues, reflecting a standardized, gendered marketing strategy in emerging markets [36].

Older adolescents were also more likely to use tobacco compared to their younger counterparts. This age gradient is plausible because older adolescents generally have greater autonomy, more disposable income or work-related access to money, wider peer networks, and more exposure to environments in which tobacco is available or socially visible [34,37,38]. They may also have had more time and opportunities to experiment, progress from trial to repeated use, and sustain tobacco use over time [25]. Adolescents who were in school had lower odds of tobacco use, particularly smoked tobacco use in Kenya and the DRC. This association may reflect the protective role of school attendance through structured daily routines, ongoing exposure to health education, adult supervision, peer norms that discourage risky behavior, and school policies restricting tobacco use on school premises [39]. By contrast, adolescents who are out of school may have fewer protective institutional supports and may be more exposed to work environments, social spaces, and peer networks in which tobacco use is more accessible or normalized. Because our out-of-school category was based on current enrolment rather than educational attainment, these findings should be interpreted as differences associated with current school enrolment rather than evidence of school dropout or school disengagement.

Parental education showed varying associations across countries. In Kenya, higher household head education (secondary level or above) was associated with lower odds of adolescent smokeless tobacco use consistent with evidence that parental education shapes health awareness and discourages risky behaviors [40,41]. By contrast, in Nigeria and the DRC, higher household head education was linked to greater odds of adolescent’s tobacco use. This contrasts with the pattern commonly observed in high-income countries, where tobacco use is more common among lower socioeconomic status [42]. One possible explanation is that these countries may represent an earlier stage of the tobacco epidemic, where tobacco use may still be more common among relatively advantaged groups. In such contexts, educated household heads often have higher incomes or social mobility, potentially fostering environments where tobacco is more accessible or normalized. In Nigeria, cultural practices such as offering cigarettes during ceremonies including marriage dowries and burials may also reinforce tobacco use among adolescents [43,44].

Environmental exposures were strongly associated with tobacco use. In DRC and Nigeria, exposure to smoking in school buildings, public transport, universities or health facilities increased the likelihood of any tobacco use. These findings highlight the influence of social and observational learning on adolescent behaviors, consistent with social cognitive theory, which suggests that young people emulate behaviors they observe and perceive as acceptable [45]. Seeing others smoke in public settings may weaken anti-tobacco norms, increase curiosity, and normalize experimentation among adolescents. Family context also played an important role. Having both parents alive was associated with lower odds of tobacco use in Nigeria and the DRC, likely due to greater supervision and emotional support. Adolescents who had lost both parents or one parent, respectively, may be more vulnerable to stress, reduced supervision, or economic hardship, all of which can increase susceptibility to risky behaviors such as tobacco use [46,47].

### Strengths and limitations

Key strengths of this study include the use of large, nationally representative samples of adolescents across three countries, and the use of a harmonized methodology that allows for cross-country comparisons. The inclusion of both in-school and out-of-school adolescents addresses a major gap in previous research. However, a small proportion of adolescents (0.3%) had completed secondary school but were not enrolled in further education. These adolescents were included within the out-of-school category because school status was classified according to current enrolment. Although this subgroup was too small for separate analysis, its inclusion may have introduced minor heterogeneity within the out-of-school category. In addition, reliance on self-reported data may have introduced social desirability or recall bias, particularly among girls and younger adolescents. Despite efforts to ensure privacy and confidentiality during data collection, under-reporting cannot be ruled out. Finally, the study did not include children who could not be linked to identifiable ‘households’, as defined for the study, e.g., those who are homeless and live on the streets.

### Implications for policy and research

These findings highlight the need for early, gender-sensitive prevention strategies that target both boys and girls, given widening industry marketing. The contrasting associations of parental education and adolescent tobacco use across countries underscore the importance of context-specific interventions, for instance, education-based messaging may be effective in Kenya, while in Nigeria and the DRC where higher parental education was linked with greater tobacco use, approaches must also address cultural norms and household practices that shape adolescent behavior. The strong association between environmental exposure and tobacco use in DRC and Nigeria points to the urgency of enforcing comprehensive smoke-free policies in schools, public transport, and public spaces. Strengthening family support and protection programs for orphans and vulnerable children can also reduce tobacco use. Finally, although our survey captured adolescents based on current school enrolment, future surveillance efforts should also seek to capture highly vulnerable groups such as homeless adolescents, who may not be reached through standard household-based surveys to ensure more inclusive tobacco prevention and policy responses.

### Conclusion

This study highlights significant cross-country differences in adolescent tobacco use, with the highest prevalence in the DRC, compared to Nigeria and Kenya. Across all countries, tobacco use was more common among boys and older adolescents and was shaped by family structure and environmental exposures in Nigeria and the DRC. In Kenya, adolescents who were currently attending school had significantly lower odds of using tobacco compared with adolescents who were not enrolled in school. While Kenya and Nigeria may have gained from earlier policy action, all countries face implementation gaps and opportunities for strengthening adolescent-focused tobacco control efforts. These findings emphasize the urgent need for context-specific adolescent centered tobacco control strategies to protect young people and reduce the future burden of tobacco related disease in sub-Saharan Africa.

## Supporting information

S1 Checklist Inclusivity in Global Research Questionnaire. (DOCX)
