# Supplementary material for: Prevalence and associated factors of adolescent tobacco use in three Sub-Saharan African countries: A comparative analysis of national cross-sectional surveys
Source: PLoS One. 2026 Aug 31;21(8):e0356891. doi: 10.1371/journal.pone.0356891 (PMC13529220; doi:10.1371/journal.pone.0356891)
Supplement: Supplementary file 2 [file pone.0356891.s002.docx]

# General comments

This is an important piece of research when considering the rise of e-cigarettes and vaping products flooding the SSA market recently, in addition to regular tobacco products. The paper is generally well written; however, it could use some edits to improve structure and flow.

# Abstract

*Background*

- The abstract is generally well written and discusses key points
- I find that the background is lacking on the “why”: take for instance obesity, high BP are also growing health concerns, what singles out tobacco use? Especially now that vaping and e-cigarette packs and regular tobacco products contain health warnings??

*Methods*

- Following my point on the ‘why’, I’d have to ask why the 10 – 17 age group was singled out in the study; what does excessive tobacco use, do to that age group?

# Introduction

- The introduction is overly simplistic and needs some restructuring; tobacco related deaths does not really tell me anything. Line 64 - 69
- With a re-iteration on my previous point as to the “Why” of this paper, what story are you telling me about tobacco use among adolescents? i.e Why should I care that young people are using tobacco products in these countries?
- I suggest following the use of structured flow and topic sentences, with a comprehensive literature review i.e. problem of tobacco use worldwide (prevalence of at least two or three specific tobacco-use related diseases) 🡪 tobacco use among adolescents (Key prognostic factors related to health as adults)🡪 literature in LMICs 🡪 literature in contexts (Kenya, Nigeria, DRC) 🡪 Gap 🡪 key study objectives to address this gap

# Methods

Study design and setting

- Although are contained in the protocol, it is important to further elaborate on the tool used; What is a harmonized questionnaire?? Who developed it, where was it tested? Was it validated? Is it an amalgamation of different tools compiled as one? If it was what were the tools that constituted the questionnaire?
- What were the standardized sampling methodologies? Some detail would make sense here too..

Measures

- It is quite important to explicitly detail the independent variable (IV) in terms of what questionnaire questions made up the variable, in addition to their internal consistencies if it applies(alpha values) and the conceptual reasons why they were included in the regression models – what the study hypothesis in terms of factors affecting tobacco use among that population?
- There is a difference between variables considered as IV and covariates, also clarify which independent variables were covariates. The IV is the "Main Character." It is the variable you are specifically interested in testing to see if it has an effect in your outcome. The covariate is a "Supporting Factor" that you aren't necessarily interested in studying, but you know it might influence the results. Were all your IVs considered covariates?

Analysis

- Well done on using the Rao-Scott correction as the weights may easily violate the independence of your variables in using representative national samples.

Results

- Please refer to PLOS formatting requirements for tables
- The descriptive tables are detailed enough (Table 1-4), the repetition on each table with the word ‘years’ seems redundant
- Were no factors found to be statistically significant in explaining the prevalence across all models? I understand your omitting the p-values considering the debate over their usefulness in research; however, they are important here considering the national representative nature of your dataset. Unless there is a prime justification as to why, you need to include a note at the bottom of the regression tables explaining the range of significance without necessarily including the actual p-values.
- And perhaps you could focus on just the statistically relevant results on your regression tables as you have done on tables 2-4. And condense tables 5-7 into one table with a focus on just the significant results
- Another suggestion is to isolate the current prevalence in the main manuscript and relegate tables 6-7 as supporting information. Still, notes on statistical significance is still important if at all you saw any in your models.

# Discussion

- It is not enough to just imply the explanations of your findings in relation to the literature, you must expand on the literature in what the authors set out to do and what they found vis a vis their methods, while contrasting it from your objectives and methods e.g lines 305 – 307 and 296 – 297 amongst other paragraphs.
