# Supplementary material for: Prevalence and associated factors of adolescent tobacco use in three Sub-Saharan African countries: A comparative analysis of national cross-sectional surveys
Source: PLoS One. 2026 Aug 31;21(8):e0356891. doi: 10.1371/journal.pone.0356891 (PMC13529220; doi:10.1371/journal.pone.0356891)
Supplement: Supplementary file 3 [file pone.0356891.s004.docx]

Dear Editors,

We thank the reviewers and editors for providing feedback to our manuscript titled “Prevalence and associated factors of adolescent tobacco use in three Sub-Saharan African countries: A comparative analysis of national cross-sectional surveys”. The authors have considered all the comments and have provided a point-by-point response to all the issues raised as detailed below.

| **Reviewers’ comments** | **Author’s response** |
| --- | --- |
| **Reviewer #1**:  Thank you for the possibility review this interesting manuscript. This is a manuscript subject matter is important for public health: the use of tobacco products among adolescents in Low- and Middle-Income Countries. The study uses national face-to-face surveys (n=18612) to investigate the prevalence and correlates of tobacco use among adolescents in Democratic Republic of Congo, Nigeria and Kenya. The results show differences in tobacco use by country and also by background variables, such as parental education and being enrolled in school. Overall, the study is relevant, easy to follow and important. I have one major suggestion and some minor comments, listed below. | Thank you for the positive, thoughtful, and helpful comments which have strengthened the manuscript. We appreciate your careful review of this manuscript. We have made the necessary corrections and as outlined below to respond to each of your comments. |
| **Major comments**  I think it would benefit if the study looked more closely on different tobacco products, not just ‘any use’ (which is also very important). Since smoked and smokeless tobacco product use has been gathered in the current surveys and the authors report these estimates, this should be also incorporated more clearly on the Abstract overall, Introduction and the Discussion sections accordingly (estimates by product categories and by ‘any use’ are reported in the Results section). This would strengthen the paper to include differences/similarities by product type since there might be differences in associated factors of smokeless and smoked tobacco. | We appreciate this valuable suggestion. We have revised the manuscript to more clearly acknowledge different tobacco product categories throughout the paper. Specifically:  *Abstract*: We clarified that the analysis considered different tobacco product categories and that results include estimates for smoked and smokeless tobacco use in addition to any tobacco use.  *Introduction*: We added text acknowledging that adolescents in these settings may be exposed to multiple tobacco products, including smoked tobacco (e.g., cigarettes and shisha) and smokeless tobacco (e.g., snuff and chewing tobacco).  *Methods***:** We expanded the Measures section to clearly define the three outcome categories used in the analysis: any tobacco use, smoked tobacco use, and smokeless tobacco use.  *Discussion***:** We have revised the interpretation of findings to explicitly reference patterns observed across both smoked and smokeless tobacco products and highlight differences and similarities in associated factors.  The Results section already presents estimates for both product categories, and the revised text now better contextualizes these findings within the broader narrative of the paper. |
| **Minor comments** |  |
| **Abstract** |  |
| Methods: response rates for each country on the Methods section. | Thank you for this suggestion. We have added the response rates for each country in the methods section of the abstract. |
| Methods: the included tobacco products could be listed. For example, currently it is unclear whether, for example, e-cigarettes are included or not (since in some countries, they are define as ‘tobacco products’ while in other countries as ‘nicotine products’). | We appreciate this clarification. The methods section of the abstract has been revised to specify the tobacco products included in the definition of tobacco use. The text now states that tobacco products included cigarettes, cigars, pipes, shisha/waterpipe, and smokeless tobacco products such as snuff and chewing tobacco. (Note: e-cigarettes were not included in the definition of tobacco products in this study.) |
| Methods: How was ‘current tobacco use’ defined? Any use during the past 30 days? | Thank you for this important clarification. We have revised the methods section of the abstract to define current tobacco use as self-reported use of any tobacco product in the past 30 days. |
| **Introduction**  Introduction is concise and easy to follow. | Thank you for this positive feedback |
| **Methods**  Out-of-school children. Is it possible that some of the older adolescents have already finished their schooling and thus not interpreted as drop-outs? Should this be taken into account in analyses for example by omitting them or classifying them in their own class? | Thank you for this observation. We have clarified this definition in the methods section. Out-of-school adolescents were defined as those who had never attended school or who had previously enrolled but were not currently attending school at the time of the survey, this could be either because they had dropped out or had completed schooling and were not enrolled in further education (e.g., college). This clarification has been added to the measures section of the manuscript. |
| “household wealth quintile.” How was this measured? | Thank you for this comment. We have clarified that the household wealth index was constructed using principal component analysis based on household assets, housing characteristics, and access to utilities. Households were then categorized into wealth quintiles. This information has been added to the methods section. See page 7, lines 144-146. |
| Were there any missing information by study variables? | Thank you for this question. Missing data across study variables were minimal (<5%). However, some responses recorded as “refused” were treated as missing values and excluded from the regression analyses using a complete-case approach. We have clarified this in the methods section of the revised manuscript. See page 8, lines 179-181 |
| Functional disability is not described in the methods section although presented in tables. | Thank you for pointing out this omission. We have now added a description of functional disability in the measures section, noting that disability status was assessed using the Washington Group Short Set of child functioning questions. See page 7, lines 151-155 |
| **Results**  Table 1. Maybe one decimal is enough for the estimates. | Thank you for your suggestion. We have carefully considered reducing the estimates in table 1 to one decimal place. However, we have retained two decimal places to ensure consistency with the presentation of all subsequent tables and to maintain a uniform level of precision across the manuscript. |
| Tables 1–4. Not all background variables (per the description in the Methos section) are included in the table, such as household wealth. Some rationale for excluding some of the factors should be explicitly presented. | Thank you for this observation. In the revised manuscript, we have added household wealth quintile to Table 1 to ensure that all key background characteristics described in the methods are represented in the descriptive table. Wealth quintile was not included in the stratified prevalence tables (Tables 2–4) to maintain clarity and readability, as these tables already present multiple subgroup comparisons. However, household wealth was included as an explanatory variable in the multivariable regression models examining factors associated with tobacco use. We have clarified this in the revised manuscript. |
| The heading ”Factors associated with tobacco use”. Before this section, some factors that are associated with tobacco use are already described. Maybe rename this section to take into account the adjusted estimates? | Thank you for this helpful suggestion. The section heading has been revised to “Adjusted multivariable factors associated with tobacco use” to clarify that the section presents adjusted estimates from regression models. See page 15, line 257 |
| “Being employed or in self-employed increased…” There is an additional “in” in the sentence. | Thank you for identifying this typographical error. The sentence has been corrected. See page 19, line 281 |
| When reporting point estimates in the text, include also corresponding 95% CI, thus the readers can assess the strength of the association. | Thank you for this suggestion. We have revised the results section to include the corresponding 95% confidence intervals alongside point estimates when reporting regression results. |
| **Discussion**  “This may reflect socioeconomic dynamics, as better educated household heads often have higher incomes or social mobility, potentially fostering environments where tobacco is more accessible or normalized” This is interesting and contradicts tobacco use trends in Western societies (see for example Thun et al. 2012 https://doi.org/10.1136/tobaccocontrol-2011-050294 ) Maybe some possible causes for this difference could be presented? | Thank you for your helpful observation. We have expanded the discussion to acknowledge that this pattern is different from what is often observed in high-income countries. We now explain that in some LMIC settings, where the tobacco epidemic may be at a different stage, households with higher education may have higher income and greater access to tobacco products and social environments where tobacco use is more visible or accepted. See page 23, lines 375-384 |
| The differences in tobacco use by school going status are interesting. These could be discussed more. | Thank you for this suggestion. We have expanded the discussion on schooling status to highlight the protective role of school attendance through structured routines, health education, supervision, and tobacco-restrictive environments, while recognizing that out-of-school adolescents may have fewer protective support and greater exposure to settings where tobacco use is more accessible or normalized. See page 22-23, lines 361-371. |
| “Finally, expanding surveillance to capture out-of-school and homeless adolescents through targeted surveys or integration into” This is a bit ambiguous as the current study included out-of-school children yet it did not include homeless children, if I am correct. | Thank you for this observation. We have revised the sentence. The text now indicates that future surveillance efforts should consider strategies to include homeless adolescents and other highly vulnerable groups who may not be reached through household-based surveys. See page 25, lines 416-420 |
| The references 29 and 30 are the same. | Thank you for identifying this error. We have corrected the duplicated reference in the revised manuscript. |
|  | |
| **Reviewer #2:**  This is an important piece of research when considering the rise of e-cigarettes and vaping products flooding the SSA market recently, in addition to regular tobacco products. The paper is generally well written; however, it could use some edits to improve structure and flow. | Thank you for your valuable and positive feedback. We appreciate your careful review of this manuscript. We have made the necessary corrections as outlined below to respond to each of your comments. |
| **Abstract**  Background  The abstract is generally well written and discusses key points  I find that the background is lacking on the “why”: take for instance obesity, high BP are also growing health concerns, what singles out tobacco use? Especially now that vaping and e-cigarette packs and regular tobacco products contain health warnings?? | Thank you for this insightful comment. We appreciate your suggestion to add the "why” to the abstract background section to better explain the 10-17 age group in the methods section. |
| Methods  Following my point on the ‘why’, I’d have to ask why the 10 – 17 age group was singled out in the study; what does excessive tobacco use, do to that age group? |  |
| **Introduction**  The introduction is overly simplistic and needs some restructuring; tobacco-related deaths does not really tell me anything. Line 64 – 69  With a re-iteration on my previous point as to the “Why” of this paper, what story are you telling me about tobacco use among adolescents? i.e Why should I care that young people are using tobacco products in these countries?  I suggest following the use of structured flow and topic sentences, with a comprehensive literature review i.e. problem of tobacco use worldwide (prevalence of at least two or three specific tobacco-use related diseases) 🡪 tobacco use among adolescents (Key prognostic factors related to health as adults) 🡪 literature in LMICs 🡪 literature in contexts (Kenya, Nigeria, DRC) 🡪 Gap 🡪 key study objectives to address this gap | Thank you for this helpful suggestion. We have revised and reorganized the introduction to improve its structure and narrative flow. The revised introduction now follows a clearer progression: the global burden of tobacco use and its health consequences, the importance of adolescence as a critical period for tobacco initiation, the growing concern of tobacco use in LMICs and sub-Saharan Africa, limitations of existing data sources such as school-based surveys, the vulnerability of out-of-school adolescents, and the evidence gap in nationally representative data from countries such as the DRC, Nigeria, and Kenya. |
| **Methods**  Study design and setting  Although are contained in the protocol, it is important to further elaborate on the tool used; What is a harmonized questionnaire?? Who developed it, where was it tested? Was it validated? Is it an amalgamation of different tools compiled as one? If it was what were the tools that constituted the questionnaire? | Thank you for this helpful suggestion. We have expanded the methods section to clarify that the harmonized questionnaire was developed as part of the DaYTA program, informed by established tobacco surveillance instruments including the Global Youth Tobacco Survey (GYTS), GHSH, NYTS among others. We also indicate that the tool was reviewed by experts and piloted during field testing to ensure clarity and consistency across countries. See page 5, lines 103-111 |
| What were the standardized sampling methodologies? Some detail would make sense here too.. | Thank you for this comment. We have expanded the description of the sampling methodology to indicate that a multistage stratified cluster sampling design was used, including selection of regions, enumeration areas using probability proportional to size, and systematic sampling of households within clusters. See page 5-6, lines 112-118 |
| Measures  It is quite important to explicitly detail the independent variable (IV) in terms of what questionnaire questions made up the variable, in addition to their internal consistencies if it applies(alpha values) and the conceptual reasons why they were included in the regression models – what the study hypothesis in terms of factors affecting tobacco use among that population?. There is a difference between variables considered as IV and covariates, also clarify which independent variables were covariates. The IV is the "Main Character." It is the variable you are specifically interested in testing to see if it has an effect in your outcome. The covariate is a "Supporting Factor" that you aren't necessarily interested in studying, but you know it might influence the results. Were all your IVs considered covariates? | Thanks for the comment. We agree that distinguishing between primary independent variables and covariates can improve clarity, particularly when a study is designed to test specific hypotheses. In our analysis, however, the modelling approach was to assess the effect of each variable while controlling for the others. Therefore, all variables included in the regression models were treated as predictors (explanatory variables) without a priori classification into primary independent variables and covariates. We have also clarified this in the measures and statistical analysis section to avoid ambiguity and improve clarity. |
| **Analysis**  Well done on using the Rao-Scott correction as the weights may easily violate the independence of your variables in using representative national samples. | Thank you for your positive feedback. |
| **Results**  Please refer to PLOS formatting requirements for tables | Thank you for this suggestion. We have reviewed the tables and revised their formatting to align with PLOS ONE table formatting requirements. |
| The descriptive tables are detailed enough (Table 1-4), the repetition on each table with the word ‘years’ seems redundant | Thank you for this observation. We have revised the tables to remove the repeated use of the word “years” in the age categories to improve readability (age categories are now presented as 10–12, 13–15, and 16–17). |
| Were no factors found to be statistically significant in explaining the prevalence across all models? I understand your omitting the p-values considering the debate over their usefulness in research; however, they are important here considering the national representative nature of your dataset. Unless there is a prime justification as to why, you need to include a note at the bottom of the regression tables explaining the range of significance without necessarily including the actual p-values. | Thank you for raising this important point. Several factors were statistically significant in the regression models. Rather than presenting p-values for each coefficient, we report adjusted odds ratios with corresponding 95% confidence intervals, which provide information on both the magnitude and statistical significance of associations. To further clarify this, we have added a note to the regression tables indicating that associations are considered statistically significant when the 95% confidence interval does not include 1.0. |
| And perhaps you could focus on just the statistically relevant results on your regression tables as you have done on tables 2-4. And condense tables 5-7 into one table with a focus on just the significant results | Thank you for this suggestion. We have combined tables 5-7 into a single table to improve clarity and streamline presentation. We have revised the results section to focus primarily on statistically significant associations, while retaining the full regression outputs in the table for completeness and transparency. |
| Another suggestion is to isolate the current prevalence in the main manuscript and relegate tables 6-7 as supporting information. Still, notes on statistical significance is still important if at all you saw any in your models. | We appreciate this suggestion. Because smoked and smokeless tobacco represent distinct patterns of tobacco use and were central to the comparative analysis, we have chosen to retain these results in the main manuscript. However, we have streamlined the presentation in the results section to focus on key findings while keeping the detailed regression table available for readers. |
| **Discussion**  It is not enough to just imply the explanations of your findings in relation to the literature, you must expand on the literature in what the authors set out to do and what they found vis a vis their methods, while contrasting it from your objectives and methods e.g lines 305 – 307 and 296 – 297 amongst other paragraphs. | Thank you for this important comment. We have revised the discussion to provide fuller interpretation of the findings in relation to both prior literature and the objectives of the study. Specifically, we expanded the discussion of the age pattern in tobacco use by explaining why older adolescents may be at increased risk, we have also elaborated on the role of schooling status as a protective factor, and provided a clearer interpretation of the positive association between higher household head education and adolescent tobacco use in Nigeria and the DRC. We have further strengthened the discussion of environmental exposure and family context in relation to the study’s household-based, comparative design. |
| Lines 305–307 and 296–297 need more development. | Thank you for this clarification. We have expanded these parts of the discussion. The text on older adolescents now explains possible mechanisms such as greater autonomy, broader peer networks, increased access to money, and longer exposure windows for experimentation. The text on Nigeria’s ceremonial use of cigarettes has also been contextualized within broader social visibility and normalization of tobacco in some settings. See page 22-23, lines 356-361, and page 23 lines 375-384 |

Sincerely,

The corresponding author
