# Supplementary material for: Prevalence and associated factors of adolescent tobacco use in three Sub-Saharan African countries: A comparative analysis of national cross-sectional surveys
Source: PLoS One. 2026 Aug 31;21(8):e0356891. doi: 10.1371/journal.pone.0356891 (PMC13529220; doi:10.1371/journal.pone.0356891)
Supplement: Supplementary file 4 [file pone.0356891.s005.docx]

**Response to Reviewers**

We sincerely thank the academic editor and reviewers for their careful evaluation of our manuscript and for the constructive comments provided. We have revised the manuscript in response to every comment. Below we provide a point-by-point response describing how each issue has been addressed in the revised manuscript.

| **Reviewers’ comments** | **Author’s response** |
| --- | --- |
| **Academic editor:**  The categorization of adolescents who completed schooling together with school dropouts under the “out-of-school” category requires stronger justification or additional analyses, as these groups may differ substantially in age and exposure to health education. | Thank you for this important comment. We agree that adolescents who had completed secondary school may differ from those who had never attended school or had dropped out. School status in our study was defined according to current enrolment in formal education rather than educational attainment. Adolescents who had completed secondary school but were not enrolled in college or any other educational institution represented only 0.3% of the pooled study population. Given the very small size of this subgroup, separate analyses would have produced unstable estimates and limited interpretability. We therefore retained them within the out-of-school category to reflect non-enrolment at the time of the survey. We have strengthened the justification in the Methods, revised the Discussion to avoid interpreting the findings as reflecting 'school disengagement', and acknowledged this classification as a study limitation. |
| The description of outcome variables (any tobacco use, smoked tobacco use and smokeless tobacco use) should be made more explicit throughout the Abstract and Methods. | Thank you for this helpful suggestion. We revised both the Abstract and Methods to state that the study examined three outcome variables: current use of any tobacco, smoked tobacco, and smokeless tobacco. We also clarified that separate multivariable logistic regression models were fitted for each outcome. |
| Some statements in the Discussion should be moderated to avoid causal language and claims regarding temporal trends. | Thank you. We revised the Discussion to avoid causal language by replacing 'influence' with 'association'. We have also moderated statements relating to temporal trends and now state that our findings are broadly consistent with WHO-reported declines in adolescent tobacco use rather than implying that our study assessed trends. |
| Minor terminology, reference group and typographical errors should be corrected. | Thank you. We carefully proofread the manuscript, corrected typographical and formatting errors, clarified reference groups where appropriate, and improved consistency of terminology throughout. |
|  | |
| **Reviewer #1:**  **Major Comment**  Out-of-school children. Based on the definition, out-of-school children include those who have completed school (vs. dropped out). A strong rationale for pooling these with those who have dropped-out should be give. At least in Western context, these groups would be very different and pooling them together would possibly give biased estimates that would mask the differences of the two groups. Also, age of the respondent would be highly important here, as age would be highly correlated with having completed education. Somehow this also should be taken into account. Furthermore, again at least in the Western context, pooling those who have completed school with school-going children rather than those who have dropped out would make more sense, although this also would be a bit problematic. Thus, one possibility would be to omit those who have completed schooling from the analyses or treating them as one separate category (although age should be somehow taken into account then, currently age has been adjusted for if I’m not mistaken, which is one option), or pooling them with another category (as is has been currently done for those who have dropped out). All of these options would need much stronger rationalization than has been done currently. The need for this is highlighted, for example, in Discussion section where it is stated that: “Adolescents who were in school had lower odds of tobacco use, particularly smoked tobacco use in Kenya and the DRC. This may reflect the protective role of school attendance through structured daily routines, exposure to health education, adult supervision, peer norms that discourage risky behavior, and school policies restricting tobacco use on school premises.” Wouldn’t those who had completed education be exposed to health education similarly than those in-school? These could be exposed to health education even more than those currently in school. Whereas those who had dropped out would potentially be less exposed to health education. Also, the authors state that: “the association observed here is consistent with broader evidence that school disengagement is linked to increased vulnerability to risk behaviors, including tobacco use”. Could this be said of individuals who have completed schooling? Could they be described as persons with ‘school disengagement’? | Thank you for this thoughtful and constructive comment. We agree that adolescents who had completed secondary school may differ from those who had never attended school or had dropped out. In our study, however, school status was defined according to current enrolment in formal education at the time of the survey rather than educational attainment, as the survey collected information on current school enrolment. Our analysis was therefore to examine the association between current enrolment in formal education and tobacco use. Consequently, adolescents who had completed secondary school but were not enrolled in college or any other educational institution at the time of data collection were classified as out-of-school because they were no longer participating in formal education.  Adolescents who had completed secondary school represented only 0.3% of the pooled study population. Given the very small size of this subgroup, analysing them as a separate category would have produced unstable estimates with limited statistical interpretability. Also, because this subgroup constituted such a small proportion of the sample, retaining them within the out-of-school category is unlikely to have materially influenced the observed associations.  We also acknowledge the reviewer's point that adolescents who have completed school may differ from those who have dropped out, particularly regarding prior exposure to health education and school environments. As the reviewer noted, age is closely related to school completion; accordingly, age was included as a covariate in all adjusted analyses to account for this potential confounding. To further address this concern, we have strengthened the justification for this classification in the Methods section, revised the Discussion to avoid interpreting our findings in terms of "school disengagement," and clarified that the observed associations should be interpreted as differences between adolescents who were and were not enrolled in formal education at the time of the survey, rather than differences attributable specifically to school dropout. Finally, we have acknowledged this classification as a limitation in the Strengths and Limitations section. |
| Abstract should include all the used dependent variables, not just "any use" (methods and results), as this relates to the contribution the paper makes on the literature. | Thank you. We revised the Abstract to state that the analyses examined three outcomes; current use of any tobacco, smoked tobacco, and smokeless tobacco. |
| Introduction: Somewhat strong emphasis has been given to describe out of school children's tobacco use. Was the aim of the study to compare out of school children's and school going children's tobacco use? Currently, the text proposes so yet the results and discussion section do not concentrate on this difference that much. | Thank you. We revised the Introduction to emphasize the broader evidence gap in nationally representative data including both in-school and out-of-school adolescents, and repositioned inclusion of out-of-school adolescents as a strength of the study rather than its primary focus. |
| Introduction: “To fill this gap...” The gap could be described in a sentence preceding this sentence, making the readability, enhancing the readability of the text. | Thank you. We added a sentence immediately before the study objective explaining that existing school-based surveillance excludes adolescents who are not attending school, limiting nationally representative estimates and identification of vulnerable groups. |
| Study design and setting: The section could be clarified to describe one theme at a time. Currently, the data collection method has been described in two places (2nd paragraph and 3rd paragraph, see texts including “enumeration areas”). This could be done by moving the following sentence later on in the paragraph/text: “Written informed consent was obtained from parents or guardians of all participating minors, and written assent was obtained from adolescents prior to data collection.” | Thank you. We’ve reorganized the Study Design and Setting section so that sampling and data collection are presented together, followed by country-specific implementation details, and moved the informed consent statement to improve the logical flow. |
| Statistical analyses: The authors have currently, based on the reviewer suggestions, to included both smoking and smokeless tobacco use as dependent variables more explicitly. However, on the analyses section, a more explicit description of how these variables were used should still be provided. Currently it is stated that: “current tobacco use served as the outcome variable” whereas, if I understand correctly, there are three different dependent variables (any tobacco use, smoking and smokeless tobacco use). | Thank you. We clarified that separate logistic regression models were fitted for each country and for each of the three outcomes and described how explanatory variables were incorporated for each model. |
| Analyses, “a set of explanatory variables representing comprising individual-, household-, and environmental- level factors were included in the regression models to ensure comparability across countries." I am not sure I agree with this sentence. Rather than adjusting for the similar variables (which is also relevant), for example, the similar data collection procedures and the target population in addition to the actual respondent characteristics make the comparison possible. | Thank you. We revised the text to clarify that explanatory variables were included to adjust for potential confounding and facilitate consistent comparison of associations across countries rather than to ensure comparability itself. |
| The text could be read for typos once again. There are typos on Table 5 (‘Snokeless’ instead of ‘Smokeless’) and in the Results section: “in Kenya, and decreased odds in the in DRC for smoked tobacco...”. | Thank you. We have corrected the misspelling 'Snokeless', removed the duplicated word 'the', and undertook an additional proofread of the manuscript. |
| Discussion, “Overall, the findings align with evidence of a declining trend in adolescent tobacco use across Africa over the past decade”. This study did not examine or report trends/changes in tobacco use, so this sentence could be toned down. | Thank you. We’ve revised the statement to read that our findings are broadly consistent with WHO-reported declines in adolescent tobacco use. |
| Discussion, “Although prevalence was highest for any tobacco use, product-specific patterns revealed important differences.” The prevalence for any tobacco use is by definition higher than for each product (smoked, smokeless), thus, this sentence could be revised to taken this into account. | Thank you. We revised the sentence to acknowledge that the higher prevalence of any tobacco use was expected while emphasizing that product-specific analyses revealed important differences in patterns of use. |
| Discussion, “semi-orphaned adolescents”. The term should be defined. | Thank you for this helpful comment. We revised the Discussion to replace the term "semi-orphaned adolescents" with descriptive wording, referring to adolescents who had lost one parent, to improve clarity and ensures that the text is understandable to a broad readership. |
| Discussion, “The strong influence of environmental exposure in DRC and Nigeria points to the urgency…” Please, avoid causal language (influence) and use more appropriate terms that take into account the current study design (is associated with, etc.) | Thank you. We replaced causal wording throughout the Discussion with language appropriate for a cross-sectional study. |
| Conclusion, “In Kenya, adolescents who were attending school had significantly lower odds of using tobacco.” Please, include the reference group here for clarity. | Thank you. We now specify that adolescents attending school had lower odds of tobacco use compared with adolescents who were not enrolled in school. |
|  | |
| **Reviewer #2:**  Good job in addressing the comments. I have no further addendum to the structure and content of the manuscript. | Thank you for the positive assessment of our revised manuscript and for the constructive comments provided throughout the review process. |
