# Supplementary material for: Prevalence and associated factors of adolescent tobacco use in three Sub-Saharan African countries: A comparative analysis of national cross-sectional surveys
Source: PLoS One. 2026 Aug 31;21(8):e0356891. doi: 10.1371/journal.pone.0356891 (PMC13529220; doi:10.1371/journal.pone.0356891)
Supplement: Peer review history

# Peer review: Decision Letter 0

**DOI:** 10.1371/journal.pone.0356891.r001

**Authors:** Bijit Biswas

5 Mar 2026

Dear Dr. Kyule,

Thank you for submitting your manuscript to PLOS ONE. After careful consideration, we feel that it has merit but does not fully meet PLOS ONE’s publication criteria as it currently stands. Therefore, we invite you to submit a revised version of the manuscript that addresses the points raised during the review process.

==============================

**ACADEMIC EDITOR:**  The manuscript addresses an important public health issue and is generally well conducted. However, the paper would benefit from clearer reporting of tobacco product categories (smoked vs. smokeless) across the Abstract, Introduction, and Discussion. Minor clarifications regarding methods (definitions of current use, response rates, wealth measurement, and missing data) and improved presentation of tables and estimates would further strengthen the manuscript.

==============================

Please submit your revised manuscript by Apr 19 2026 11:59PM. If you will need more time than this to complete your revisions, please reply to this message or contact the journal office at plosone@plos.org. When you're ready to submit your revision, log on to https://www.editorialmanager.com/pone/ and select the 'Submissions Needing Revision' folder to locate your manuscript file.

- A letter that responds to each point raised by the academic editor and reviewer(s). You should upload this letter as a separate file labeled 'Response to Reviewers'.
- A marked-up copy of your manuscript that highlights changes made to the original version. You should upload this as a separate file labeled 'Revised Manuscript with Track Changes'.
- An unmarked version of your revised paper without tracked changes. You should upload this as a separate file labeled 'Manuscript'.

If applicable, we recommend that you deposit your laboratory protocols in protocols.io to enhance the reproducibility of your results. Protocols.io assigns your protocol its own identifier (DOI) so that it can be cited independently in the future. For instructions see: https://journals.plos.org/plosone/s/submission-guidelines#loc-laboratory-protocols. Additionally, PLOS ONE offers an option for publishing peer-reviewed Lab Protocol articles, which describe protocols hosted on protocols.io. Read more information on sharing protocols at https://plos.org/protocols?utm_medium=editorial-email&utm_source=authorletters&utm_campaign=protocols.

We look forward to receiving your revised manuscript.

Kind regards,

Bijit Biswas, MBBS, MD, DNB

Academic Editor

PLOS One

Journal Requirements:

When submitting your revision, we need you to address these additional requirements.

1. Please ensure that your manuscript meets PLOS ONE's style requirements, including those for file naming. The PLOS ONE style templates can be found at

https://journals.plos.org/plosone/s/file?id=wjVg/PLOSOne_formatting_sample_main_body.pdf and https://journals.plos.org/plosone/s/file?id=ba62/PLOSOne_formatting_sample_title_authors_affiliations.pdf

2.  Please include a complete copy of PLOS’ questionnaire on inclusivity in global research in your revised manuscript. Our policy for research in this area aims to improve transparency in the reporting of research performed outside of researchers’ own country or community. The policy applies to researchers who have travelled to a different country to conduct research, research with Indigenous populations or their lands, and research on cultural artefacts. The questionnaire can also be requested at the journal’s discretion for any other submissions, even if these conditions are not met. Please find more information on the policy and a link to download a blank copy of the questionnaire here: https://journals.plos.org/plosone/s/best-practices-in-research-reporting. Please upload a completed version of your questionnaire as Supporting Information when you resubmit your manuscript.

3. Your ethics statement should only appear in the Methods section of your manuscript. If your ethics statement is written in any section besides the Methods, please delete it from any other section.

4. In the online submission form, you indicated that the dataset generated and analyzed during this study will be publicly accessible on the Tobacco Control Data Initiative (TCDI) dashboard (https://tobaccocontroldata.org) from December 1, 2025. Prior to this date, the dataset may be made available from the authors upon reasonable request.

All PLOS journals now require all data underlying the findings described in their manuscript to be freely available to other researchers, either 1. In a public repository, 2. Within the manuscript itself, or 3. Uploaded as supplementary information.

This policy applies to all data except where public deposition would breach compliance with the protocol approved by your research ethics board. If your data cannot be made publicly available for ethical or legal reasons (e.g., public availability would compromise patient privacy), please explain your reasons on resubmission and your exemption request will be escalated for approval.

5. When completing the data availability statement of the submission form, you indicated that you will make your data available on acceptance. We strongly recommend all authors decide on a data sharing plan before acceptance, as the process can be lengthy and hold up publication timelines. Please note that, though access restrictions are acceptable now, your entire data will need to be made freely accessible if your manuscript is accepted for publication. This policy applies to all data except where public deposition would breach compliance with the protocol approved by your research ethics board. If you are unable to adhere to our open data policy, please kindly revise your statement to explain your reasoning and we will seek the editor's input on an exemption. Please be assured that, once you have provided your new statement, the assessment of your exemption will not hold up the peer review process.

6. If the reviewer comments include a recommendation to cite specific previously published works, please review and evaluate these publications to determine whether they are relevant and should be cited. There is no requirement to cite these works unless the editor has indicated otherwise.

7. Please review your reference list to ensure that it is complete and correct. If you have cited papers that have been retracted, please include the rationale for doing so in the manuscript text, or remove these references and replace them with relevant current references. Any changes to the reference list should be mentioned in the rebuttal letter that accompanies your revised manuscript. If you need to cite a retracted article, indicate the article’s retracted status in the References list and also include a citation and full reference for the retraction notice.

Additional Editor Comments :

The manuscript addresses an important public health issue and is generally well conducted. However, the paper would benefit from clearer reporting of tobacco product categories (smoked vs. smokeless) across the Abstract, Introduction, and Discussion. Minor clarifications regarding methods (definitions of current use, response rates, wealth measurement, and missing data) and improved presentation of tables and estimates would further strengthen the manuscript.

[Note: HTML markup is below. Please do not edit.]

Reviewers' comments:

Reviewer's Responses to Questions

**Comments to the Author**

1. Is the manuscript technically sound, and do the data support the conclusions?

Reviewer #1: Yes

Reviewer #2: Yes

**********

2. Has the statistical analysis been performed appropriately and rigorously?

Reviewer #1: Yes

Reviewer #2: Yes

**********

3. Have the authors made all data underlying the findings in their manuscript fully available?

The PLOS Data policy

Reviewer #1: No

Reviewer #2: No

**********

4. Is the manuscript presented in an intelligible fashion and written in standard English?

Reviewer #1: Yes

Reviewer #2: Yes

**********

Reviewer #1: Review for ‘Prevalence and associated factors of adolescent tobacco use in three Sub-Saharan African countries: A comparative analysis of national cross-sectional surveys’ (PONE-D-25-62067).

Thank you for the possibility review this interesting manuscript. This is a manuscript subject matter is important for public health: the use of tobacco products among adolescents in Low- and Middle-Income Countries. The study uses national face-to-face surveys (n=18612) to investigate the prevalence and correlates of tobacco use among adolescents in Democratic Republic of Congo, Nigeria and Kenya. The results show differences in tobacco use by country and also by background variables, such as parental education and being enrolled in school. Overall, the study is relevant, easy to follow and important. I have one major suggestion and some minor comments, listed below.

Major comments

1. I think it would benefit if the study looked more closely on different tobacco products, not just ‘any use’ (which is also very important). Since smoked and smokeless tobacco product use has been gathered in the current surveys and the authors report these estimates, this should be also incorporated more clearly on the Abstract overall, Introduction and the Discussion sections accordingly (estimates by product categories and by ‘any use’ are reported in the Results section). This would strengthen the paper to include differences/similarities by product type since there might be differences in associated factors of smokeless and smoked tobacco.

Minor comments

Abstract

2. Methods: response rates for each country on the Methods section.

3. Methods: the included tobacco products could be listed. For example, currently it is unclear whether, for example, e-cigarettes are included or not (since in some countries, they are define as ‘tobacco products’ while in other countries as ‘nicotine products’).

4. Methods: How was ‘current tobacco use’ defined? Any use during the past 30 days?

Introduction

5. Introduction is concise and easy to follow.

Methods

6. Out-of-school children. Is it possible that some of the older adolescents have already finished their schooling and thus not interpreted as drop-outs? Should this be taken into account in analyses for example by omitting them or classifying them in their own class?

7. “household wealth quintile.” How was this measured?

8. Were there any missing information by study variables?

9. Functional disability is not described in the Methods section although presented in tables.

Results

10. Table 1. Maybe one decimal is enough for the estimates.

11. Tables 1–4. Not all background variables (per the description in the Methos section) are included in the table, such as household wealth. Some rationale for excluding some of the factors should be explicitly presented.

12. The heading ”Factors associated with tobacco use”. Before this section, some factors that are associated with tobacco use are already described. Maybe rename this section to take into account the adjusted estimates?

13. “Being employed or in self-employed increased…” There is an additional “in” in the sentence.

14. When reporting point estimates in the text, include also corresponding 95% CI, thus the readers can assess the strength of the association.

Discussion

15. “This may reflect socioeconomic dynamics, as better educated household heads often have higher incomes or social mobility, potentially fostering environments where tobacco is more accessible or normalized” This is interesting and contradicts tobacco use trends in Western societies (see for example Thun et al. 2012 https://doi.org/10.1136/tobaccocontrol-2011-050294 ) Maybe some possible causes for this difference could be presented?

16. The differences in tobacco use by school going status are interesting. These could be discussed more.

17. “Finally, expanding surveillance to capture out-of-school and homeless adolescents through targeted surveys or integration into” This is a bit ambiguous as the current study included out-of-school children yet it did not include homeless children, if I am correct.

18. The references 29 and 30 are the same.

Reviewer #2: This is an important piece of research when considering the rise of e-cigarettes and vaping products flooding the SSA market recently, in addition to regular tobacco products. The paper is generally well written; however, it could use some edits to improve structure and flow.

**********

what does this mean?). If published, this will include your full peer review and any attached files.

If you choose “no”, your identity will remain anonymous but your review may still be made public.

**Do you want your identity to be public for this peer review?**  For information about this choice, including consent withdrawal, please see our Privacy Policy

Reviewer #1: No

Reviewer #2: No

**********

[NOTE: If reviewer comments were submitted as an attachment file, they will be attached to this email and accessible via the submission site. Please log into your account, locate the manuscript record, and check for the action link "View Attachments". If this link does not appear, there are no attachment files.]

To ensure your figures meet our technical requirements, please review our figure guidelines: https://journals.plos.org/plosone/s/figures

You may also use PLOS’s free figure tool, NAAS, to help you prepare publication quality figures: https://journals.plos.org/plosone/s/figures#loc-tools-for-figure-preparation.

NAAS will assess whether your figures meet our technical requirements by comparing each figure against our figure specifications.

Attachment Submitted filename: General-Specific comments.docx

# Author comment: Author response to Decision Letter 1

**DOI:** 10.1371/journal.pone.0356891.r002

17 Apr 2026

We have revised the manuscript to address all comments from the academic editor and reviewers. A detailed response is provided in the “Response to Reviewers” document, and all requested files have been uploaded.

Attachment Submitted filename: Response to Reviewers.docx

# Peer review: Decision Letter 1

**DOI:** 10.1371/journal.pone.0356891.r003

**Authors:** Bijit Biswas

9 Jun 2026

Dear Dr. Grace Nduku Kyule,

Thank you for submitting your manuscript to PLOS ONE. After careful consideration, we feel that it has merit but does not fully meet PLOS ONE’s publication criteria as it currently stands. Therefore, we invite you to submit a revised version of the manuscript that addresses the points raised during the review process.

==============================

**ACADEMIC EDITOR:**  Thank you for the revision. The manuscript has improved considerably and addresses an important public health issue. However, several points still require attention. In particular, the categorization of adolescents who completed schooling together with school dropouts under the “out-of-school” category requires stronger justification or additional analyses, as these groups may differ substantially in age and exposure to health education. The description of outcome variables (any tobacco use, smoked tobacco use, and smokeless tobacco use) should be made more explicit throughout the Abstract and Methods. Some statements in the Discussion should be moderated to avoid causal language and claims regarding temporal trends, and minor issues related to terminology, reference groups, and typographical errors should be corrected. Overall, these revisions are feasible and would further strengthen the manuscript.

==============================

Please submit your revised manuscript by Jul 24 2026 11:59PM. If you will need more time than this to complete your revisions, please reply to this message or contact the journal office at plosone@plos.org. When you're ready to submit your revision, log on to https://www.editorialmanager.com/pone/ and select the 'Submissions Needing Revision' folder to locate your manuscript file.

- A letter that responds to each point raised by the academic editor and reviewer(s). You should upload this letter as a separate file labeled 'Response to Reviewers'.
- A marked-up copy of your manuscript that highlights changes made to the original version. You should upload this as a separate file labeled 'Revised Manuscript with Track Changes'.
- An unmarked version of your revised paper without tracked changes. You should upload this as a separate file labeled 'Manuscript'.

If you would like to make changes to your financial disclosure, please include your updated statement in your cover letter. Guidelines for resubmitting your figure files are available below the reviewer comments at the end of this letter.

If applicable, we recommend that you deposit your laboratory protocols in protocols.io to enhance the reproducibility of your results. Protocols.io assigns your protocol its own identifier (DOI) so that it can be cited independently in the future. For instructions see: https://journals.plos.org/plosone/s/submission-guidelines#loc-laboratory-protocols. Additionally, PLOS ONE offers an option for publishing peer-reviewed Lab Protocol articles, which describe protocols hosted on protocols.io. Read more information on sharing protocols at https://plos.org/protocols?utm_medium=editorial-email&utm_source=authorletters&utm_campaign=protocols.

As the corresponding author, your ORCID iD is verified in the submission system and will appear in the published article. PLOS supports the use of ORCID, and we encourage all coauthors to register for an ORCID iD and use it as well. Please encourage your coauthors to verify their ORCID iD within the submission system before final acceptance, as unverified ORCID iDs will not appear in the published article. *Only*  the individual author can complete the verification step; PLOS staff *cannot*  verify ORCID iDs on behalf of authors.

We look forward to receiving your revised manuscript.

Kind regards,

Bijit Biswas, MBBS, MD, DNB

Academic Editor

PLOS One

Journal Requirements:

If the reviewer comments include a recommendation to cite specific previously published works, please review and evaluate these publications to determine whether they are relevant and should be cited. There is no requirement to cite these works unless the editor has indicated otherwise.

Please review your reference list to ensure that it is complete and correct. If you have cited papers that have been retracted, please include the rationale for doing so in the manuscript text, or remove these references and replace them with relevant current references. Any changes to the reference list should be mentioned in the rebuttal letter that accompanies your revised manuscript. If you need to cite a retracted article, indicate the article’s retracted status in the References list and also include a citation and full reference for the retraction notice.

Additional Editor Comments:

Thank you for the revision. The manuscript has improved considerably and addresses an important public health issue. However, several points still require attention. In particular, the categorization of adolescents who completed schooling together with school dropouts under the “out-of-school” category requires stronger justification or additional analyses, as these groups may differ substantially in age and exposure to health education. The description of outcome variables (any tobacco use, smoked tobacco use, and smokeless tobacco use) should be made more explicit throughout the Abstract and Methods. Some statements in the Discussion should be moderated to avoid causal language and claims regarding temporal trends, and minor issues related to terminology, reference groups, and typographical errors should be corrected. Overall, these revisions are feasible and would further strengthen the manuscript.

[Note: HTML markup is below. Please do not edit.]

Reviewers' comments:

Reviewer's Responses to Questions

**Comments to the Author**

Reviewer #1: All comments have been addressed

Reviewer #2: All comments have been addressed

**********

2. Is the manuscript technically sound, and do the data support the conclusions?

Reviewer #1: Partly

Reviewer #2: Yes

**********

3. Has the statistical analysis been performed appropriately and rigorously?

Reviewer #1: No

Reviewer #2: Yes

**********

4. Have the authors made all data underlying the findings in their manuscript fully available?

The PLOS Data policy

Reviewer #1: Yes

Reviewer #2: Yes

**********

5. Is the manuscript presented in an intelligible fashion and written in standard English?

Reviewer #1: Yes

Reviewer #2: Yes

**********

Reviewer #1: Review for ‘Prevalence and associated factors of adolescent tobacco use in three Sub-Saharan African countries: A comparative analysis of national cross-sectional surveys’ (PONE-D-25-62067.R1).

Thank you for the authors for their revision on the paper. They have taken editors’ and reviewers’ comments and suggestions thoroughly into account and the paper has improved. Still, I have one major comment and some minor remarks that the authors could still consider to strengthen the paper even more.

Major comment

1. Out-of-school children. Based on the definition, out-of-school children include those who have completed school (vs. dropped out). A strong rationale for pooling these with those who have dropped-out should be give. At least in Western context, these groups would be very different and pooling them together would possibly give biased estimates that would mask the differences of the two groups. Also, age of the respondent would be highly important here, as age would be highly correlated with having completed education. Somehow this also should be taken into account. Furthermore, again at least in the Western context, pooling those who have completed school with school-going children rather than those who have dropped out would make more sense, although this also would be a bit problematic. Thus, one possibility would be to omit those who have completed schooling from the analyses or treating them as one separate category (although age should be somehow taken into account then, currently age has been adjusted for if I’m not mistaken, which is one option), or pooling them with another category (as is has been currently done for those who have dropped out). All of these options would need much stronger rationalization than has been done currently. The need for this is highlighted, for example, in Discussion section where it is stated that: “Adolescents who were in school had lower odds of tobacco use, particularly smoked tobacco use in Kenya and the DRC. This may reflect the protective role of school attendance through structured daily routines, exposure to health education, adult supervision, peer norms that discourage risky behavior, and school policies restricting tobacco use on school premises.” Wouldn’t those who had completed education be exposed to health education similarly than those in-school? These could be exposed to health education even more than those currently in school. Whereas those who had dropped out would potentially be less exposed to health education. Also, the authors state that: “the association observed here is consistent with broader evidence that school disengagement is linked to increased vulnerability to risk behaviors, including tobacco use”. Could this be said of individuals who have completed schooling? Could they be described as persons with ‘school disengagement’?

Minor comments

2. Abstract should include all the used dependent variables, not just "any use" (methods and results), as this relates to the contribution the paper makes on the literature.

3. Introduction: Somewhat strong emphasis has been given to describe out of school children's tobacco use. Was the aim of the study to compare out of school children's and school going children's tobacco use? Currently, the text proposes so yet the results and discussion section do not concentrate on this difference that much.

4. Introduction: “To fill this gap...” The gap could be described in a sentence preceding this sentence, making the readability, enhancing the readability of the text.

5. Study design and setting: The section could be clarified to describe one theme at a time. Currently, the data collection method has been described in two places (2nd paragraph and 3rd paragraph, see texts including “enumeration areas”). This could be done by moving the following sentence later on in the paragraph/text: “Written informed consent was obtained from parents or guardians of all participating minors, and written assent was obtained from adolescents prior to data collection.”

6. Statistical analyses: The authors have currently, based on the reviewer suggestions, to included both smoking and smokeless tobacco use as dependent variables more explicitly. However, on the analyses section, a more explicit description of how these variables were used should still be provided. Currently it is stated that: “current tobacco use served as the outcome variable” whereas, if I understand correctly, there are three different dependent variables (any tobacco use, smoking and smokeless tobacco use).

7. Analyses, “a set of explanatory variables representing comprising individual-, household-, and environmental- level factors were included in the regression models to ensure comparability across countries." I am not sure I agree with this sentence. Rather than adjusting for the similar variables (which is also relevant), for example, the similar data collection procedures and the target population in addition to the actual respondent characteristics make the comparison possible.

8. The text could be read for typos once again. There are typos on Table 5 (‘Snokeless’ instead of ‘Smokeless’) and in the Results section: “in Kenya, and decreased odds in the in DRC for smoked tobacco...”.

9. Discussion, “Overall, the findings align with evidence of a declining trend in adolescent tobacco use across Africa over the past decade”. This study did not examine or report trends/changes in tobacco use, so this sentence could be toned down.

10. Discussion, “Although prevalence was highest for any tobacco use, product-specific patterns revealed important differences.” The prevalence for any tobacco use is by definition higher than for each product (smoked, smokeless), thus, this sentence could be revised to taken this into account.

11. Discussion, “semi-orphaned adolescents”. The term should be defined.

12. Discussion, “The strong influence of environmental exposure in DRC and Nigeria points to the urgency…” Please, avoid causal language (influence) and use more appropriate terms that take into account the current study design (is associated with, etc.)

13. Conclusion, “In Kenya, adolescents who were attending school had significantly lower odds of using tobacco.” Please, include the reference group here for clarity.

Reviewer #2: Good job in addressing the comments. I have no further addendum to the structure and content of the manuscript.

**********

what does this mean?). If published, this will include your full peer review and any attached files.

If you choose “no”, your identity will remain anonymous but your review may still be made public.

**Do you want your identity to be public for this peer review?**  For information about this choice, including consent withdrawal, please see our Privacy Policy

Reviewer #1: No

Reviewer #2: No

**********

[NOTE: If reviewer comments were submitted as an attachment file, they will be attached to this email and accessible via the submission site. Please log into your account, locate the manuscript record, and check for the action link "View Attachments". If this link does not appear, there are no attachment files.]

To ensure your figures meet our technical requirements, please review our figure guidelines: https://journals.plos.org/plosone/s/figures

You may also use PLOS’s free figure tool, NAAS, to help you prepare publication quality figures: https://journals.plos.org/plosone/s/figures#loc-tools-for-figure-preparation.

NAAS will assess whether your figures meet our technical requirements by comparing each figure against our figure specifications.

# Author comment: Author response to Decision Letter 2

**DOI:** 10.1371/journal.pone.0356891.r004

7 Jul 2026

We sincerely thank the Academic Editor and reviewers for their careful evaluation of our manuscript and for their constructive comments. We have revised the manuscript to address all comments raised during this round of review. The revisions include strengthening the justification for the classification of out-of-school adolescents, clarifying the tobacco use outcome variables and statistical analyses, refining the Introduction and Discussion, moderating causal and temporal language, improving the presentation of the manuscript, and correcting typographical and editorial issues. A detailed point-by-point response to all comments is provided in the accompanying Response to Reviewers document, and all revisions are highlighted in the tracked-changes manuscript.

Attachment Submitted filename: Response_to_Reviewers_auresp_2.docx

# Peer review: Decision Letter 2

**DOI:** 10.1371/journal.pone.0356891.r005

**Authors:** Bijit Biswas

10 Aug 2026

Prevalence and associated factors of adolescent tobacco use in three Sub-Saharan African countries: A comparative analysis of national cross-sectional surveys

PONE-D-25-62067R2

Dear Dr. Grace Nduku Kyule,

We’re pleased to inform you that your manuscript has been judged scientifically suitable for publication and will be formally accepted for publication once it meets all outstanding technical requirements.

Within one week, you’ll receive an e-mail detailing the required amendments. When these have been addressed, you’ll receive a formal acceptance letter and your manuscript will be scheduled for publication.

An invoice will be generated when your article is formally accepted. Please note, if your institution has a publishing partnership with PLOS and your article meets the relevant criteria, all or part of your publication costs will be covered. Please make sure your user information is up-to-date by logging into Editorial Manager at Editorial Manager® and clicking the ‘Update My Information' link at the top of the page. For questions related to billing, please contact billing support.

If your institution or institutions have a press office, please notify them about your upcoming paper to help maximize its impact. If they’ll be preparing press materials, please inform our press team as soon as possible -- no later than 48 hours after receiving the formal acceptance. Your manuscript will remain under strict press embargo until 2 pm Eastern Time on the date of publication. For more information, please contact onepress@plos.org.

Kind regards,

Bijit Biswas, MBBS, MD, DNB

Academic Editor

PLOS One

Additional Editor Comments (optional):

The manuscript has been revised in response to the comments raised during peer review. Both reviewers have confirmed that their previous concerns have been adequately addressed and have no further comments.

Reviewers' comments:

Reviewer's Responses to Questions

**Comments to the Author**

Reviewer #1: All comments have been addressed

**********

2. Is the manuscript technically sound, and do the data support the conclusions?

Reviewer #1: (No Response)

**********

3. Has the statistical analysis been performed appropriately and rigorously?

Reviewer #1: (No Response)

**********

4. Have the authors made all data underlying the findings in their manuscript fully available?

The PLOS Data policy

Reviewer #1: (No Response)

**********

5. Is the manuscript presented in an intelligible fashion and written in standard English?

Reviewer #1: (No Response)

**********

Reviewer #1: (No Response)

**********

what does this mean?). If published, this will include your full peer review and any attached files.

If you choose “no”, your identity will remain anonymous but your review may still be made public.

**Do you want your identity to be public for this peer review?**  For information about this choice, including consent withdrawal, please see our Privacy Policy

Reviewer #1: No

**********

# Editor report: Acceptance letter

**DOI:** 10.1371/journal.pone.0356891.r006

**Authors:** Bijit Biswas

PONE-D-25-62067R2

PLOS One

Dear Dr. Kyule,

I'm pleased to inform you that your manuscript has been deemed suitable for publication in PLOS One. Congratulations! Your manuscript is now being handed over to our production team.

At this stage, our production department will prepare your paper for publication. This includes ensuring the following:

* All references, tables, and figures are properly cited

* All relevant supporting information is included in the manuscript submission,

* There are no issues that prevent the paper from being properly typeset

You will receive further instructions from the production team, including instructions on how to review your proof when it is ready. Please keep in mind that we are working through a large volume of accepted articles, so please give us a few days to review your paper and let you know the next and final steps.

Lastly, if your institution or institutions have a press office, please let them know about your upcoming paper now to help maximize its impact. If they'll be preparing press materials, please inform our press team within the next 48 hours. Your manuscript will remain under strict press embargo until 2 pm Eastern Time on the date of publication. For more information, please contact onepress@plos.org.

You will receive an invoice from PLOS for your publication fee after your manuscript has reached the completed accept phase. If you receive an email requesting payment before acceptance or for any other service, this may be a phishing scheme. Learn how to identify phishing emails and protect your accounts at https://explore.plos.org/phishing.

If we can help with anything else, please email us at customercare@plos.org.

Thank you for submitting your work to PLOS One and supporting open access.

Kind regards,

PLOS ONE Editorial Office Staff

on behalf of

Dr. Bijit Biswas

Academic Editor

PLOS One
